# Supplementary material for: Lipidomic Profiling and Storage-Induced Changes in Cassava Flour Using LC-MS/MS
Source: Foods. 2024 Sep 25;13(19):3039. doi: 10.3390/foods13193039 (PMC11475662; doi:10.3390/foods13193039)
Supplement: Supplementary file 1 [file foods-13-03039-s001.zip › 0.Data/data.levels.pdf]

Index	Compounds	Class I	Class II	Q1 (Da)
Lipid-B-P-0973	ADGGA((O-16:0)18:1_18:2)	GL	ADGGA	1050.81677
Lipid-B-P-0967	ADGGA((O-16:0)16:0_18:2)	GL	ADGGA	1024.80481
Lipid-B-P-0965	ADGGA((O-16:0)16:0_18:1)	GL	ADGGA	1026.82092
Lipid-B-P-0023	Cer(d18:0/24:0)	SP	Cer	652.66077
Lipid-B-P-0040	Cer(d18:1/18:1)	SP	Cer	564.53557
Lipid-B-P-0039	Cer(d18:1/16:1)	SP	Cer	536.504225
Lipid-B-P-0037	Cer(d18:1/26:0)	SP	Cer	678.676419
Lipid-B-P-0035	Cer(d18:1/24:0)	SP	Cer	650.64512
Lipid-B-P-0034	Cer(d18:1/23:0)	SP	Cer	636.629469
Lipid-B-P-0033	Cer(d18:1/22:0)	SP	Cer	622.61382
Lipid-B-P-0032	Cer(d18:1/21:0)	SP	Cer	608.598169
Lipid-B-P-0022	Cer(d18:0/22:0)	SP	Cer	624.62947
Lipid-B-P-0021	Cer(d18:0/20:0)	SP	Cer	596.59817
Lipid-B-P-0020	Cer(d18:0/18:0)	SP	Cer	568.56687
Lipid-B-P-0019	Cer(d18:0/16:0)	SP	Cer	540.535569
Lipid-B-P-0014	Cer(d18:0/24:0(2OH))	SP	Cer	668.655684
Lipid-B-P-0041	Cer(d18:1/20:1)	SP	Cer	592.566825
Lipid-B-P-0042	Cer(d18:1/22:1)	SP	Cer	620.598125
Lipid-B-P-0036	Cer(d18:1/25:0)	SP	Cer	664.660769
Lipid-B-P-0044	Cer(d18:1/26:1)	SP	Cer	676.66077
Lipid-B-P-0069	Cer(d18:2/26:3)	SP	Cer	670.6012
Lipid-B-P-0068	Cer(d18:2/18:3)	SP	Cer	558.4874
Lipid-B-P-0043	Cer(d18:1/24:1)	SP	Cer	648.62947
Lipid-B-P-0066	Cer(d18:2/24:1)	SP	Cer	646.6
Lipid-B-P-0065	Cer(d18:2/22:1)	SP	Cer	618.5824
Lipid-B-P-0064	Cer(d18:2/20:1)	SP	Cer	590.5497
Lipid-B-P-0063	Cer(d18:2/18:1)	SP	Cer	562.5159
Lipid-B-P-0067	Cer(d18:2/18:2)	SP	Cer	560.4966
Lipid-B-P-0061	Cer(d18:2/26:0)	SP	Cer	676.6
Lipid-B-P-0060	Cer(d18:2/24:0)	SP	Cer	648.6
Lipid-B-P-0059	Cer(d18:2/23:0)	SP	Cer	634.6
Lipid-B-P-0058	Cer(d18:2/22:0)	SP	Cer	620.6
Lipid-B-P-0053	Cer(d18:2/16:0)	SP	Cer	536.5
Lipid-B-P-0062	Cer(d18:2/16:1)	SP	Cer	534.4869
Lipid-B-P-0089	Cer(t18:1/23:0(2OH))	SP	Cert	668.619299
Lipid-B-P-0091	Cer(t18:1/25:0(2OH))	SP	Cert	696.650599
Lipid-B-P-0092	Cer(t18:1/26:0(2OH))	SP	Cert	710.666249
Lipid-B-P-0093	Cer(t18:1/27:0(2OH))	SP	Cert	724.6788
Lipid-B-P-0098	Cer(t17:0/24:0)	SP	Cert	654.640034
Lipid-B-P-0104	Cer(t18:0/22:0)	SP	Cert	640.530484
Lipid-B-P-0103	Cer(t18:0/18:0)	SP	Cert	584.530484
Lipid-B-P-0111	Cer(t18:1/26:0)	SP	Cert	694.671334
Lipid-B-P-0073	Cer(t18:0/18:0(2OH))	SP	Cert	600.5828
Lipid-B-P-0105	Cer(t18:0/24:0)	SP	Cert	668.655684
Lipid-B-P-0088	Cer(t18:1/22:0(2OH))	SP	Cert	654.603649
Lipid-B-P-0102	Cer(t18:0/16:0)	SP	Cert	556.530484
Lipid-B-P-0087	Cer(t18:1/20:0(2OH))	SP	Cert	626.5828
Lipid-B-P-0074	Cer(t18:0/20:0(2OH))	SP	Cert	628.5828
Lipid-B-P-0085	Cer(t18:1/16:0(2OH))	SP	Cert	570.5182
Lipid-B-P-0084	Cer(t20:0/24:0(2OH))	SP	Cert	712.681899
Lipid-B-P-0083	Cer(t18:0/26:1(2OH))	SP	Cert	710.6596
Lipid-B-P-0081	Cer(t18:0/24:1(2OH))	SP	Cert	682.634949
Lipid-B-P-0080	Cer(t18:0/26:0(2OH))	SP	Cert	712.681899

Lipid-B-P-0079	Cer(t18:0/25:0(2OH))	SP	Cert	698.666249
Lipid-B-P-0078	Cer(t18:0/24:0(2OH))	SP	Cert	684.650599
Lipid-B-P-0077	Cer(t18:0/23:0(2OH))	SP	Cert	670.631
Lipid-B-P-0076	Cer(t18:0/22:0(2OH))	SP	Cert	656.6132
Lipid-B-P-0075	Cer(t18:0/21:0(2OH))	SP	Cert	642.5828
Lipid-B-P-0110	Cer(t18:1/24:0)	SP	Cert	666.640034
Lipid-B-P-0071	Cer(t17:0/24:0(2OH))	SP	Cert	670.634949
Lipid-B-P-0072	Cer(t18:0/16:0(2OH))	SP	Cert	572.5182
Lipid-B-P-0086	Cer(t18:1/18:0(2OH))	SP	Cert	598.5828
Lipid-B-P-0106	Cer(t18:0/26:0)	SP	Cert	696.686984
Lipid-B-P-1287	Coenzyme Q10	PR	CoQ	863.69147
Lipid-B-P-1286	Coenzyme Q9	PR	CoQ	795.62878
Lipid-B-P-0325	DG(16:0_18:0)	GL	DG	614.572349
Lipid-B-P-0428	DG(18:3_18:3)	GL	DG	630.509749
Lipid-B-P-0324	DG(14:0_18:0)	GL	DG	586.541049
Lipid-B-P-0405	DG(18:1_18:3)	GL	DG	634.541049
Lipid-B-P-0403	DG(18:2_18:2)	GL	DG	634.541049
Lipid-B-P-0387	DG(18:0_18:3)	GL	DG	636.556699
Lipid-B-P-0385	DG(18:1_18:2)	GL	DG	636.556699
Lipid-B-P-0381	DG(17:1_18:2)	GL	DG	622.541049
Lipid-B-P-0379	DG(16:0_18:3)	GL	DG	608.525399
Lipid-B-P-0367	DG(18:1_20:1)	GL	DG	666.603649
Lipid-B-P-0366	DG(18:1_18:1)	GL	DG	638.572349
Lipid-B-P-0365	DG(18:0_18:2)	GL	DG	638.572349
Lipid-B-P-0362	DG(17:1_18:1)	GL	DG	624.556699
Lipid-B-P-0361	DG(16:0_18:2)	GL	DG	610.541049
Lipid-B-P-0416	DG(18:2_18:3)	GL	DG	632.525399
Lipid-B-P-0355	DG(18:1_24:0)	GL	DG	724.681899
Lipid-B-P-0360	DG(16:1_18:1)	GL	DG	610.541049
Lipid-B-P-0326	DG(14:0_20:0)	GL	DG	614.572349
Lipid-B-P-0327	DG(17:0_18:0)	GL	DG	628.587999
Lipid-B-P-0328	DG(18:0_18:0)	GL	DG	642.603649
Lipid-B-P-0330	DG(14:0_22:0)	GL	DG	642.603649
Lipid-B-P-0333	DG(20:0_18:0)	GL	DG	670.634949
Lipid-B-P-0341	DG(14:0_16:1)	GL	DG	556.494099
Lipid-B-P-0323	DG(16:0_16:0)	GL	DG	586.541049
Lipid-B-P-0345	DG(15:0_18:1)	GL	DG	598.541049
Lipid-B-P-0347	DG(16:0_18:1)	GL	DG	612.556699
Lipid-B-P-0348	DG(17:0_18:1)	GL	DG	626.572349
Lipid-B-P-0351	DG(18:0_18:1)	GL	DG	640.587999
Lipid-B-P-0353	DG(18:1_20:0)	GL	DG	668.619299
Lipid-B-P-0344	DG(16:0_17:1)	GL	DG	598.541049
Lipid-B-P-0354	DG(18:1_22:0)	GL	DG	696.650599
Lipid-B-P-1170	DGDG(16:0_18:3)	GL	DGDG	932.631049
Lipid-B-P-1171	DGDG(16:1_18:2)	GL	DGDG	932.631049
Lipid-B-P-1174	DGDG(18:0_18:3)	GL	DGDG	960.662349
Lipid-B-P-1175	DGDG(18:1_18:2)	GL	DGDG	960.662349
Lipid-B-P-1181	DGDG(18:2_18:2)	GL	DGDG	958.646699
Lipid-B-P-1166	DGDG(18:0_18:2)	GL	DGDG	962.677999
Lipid-B-P-1190	DGDG(18:2_18:3)	GL	DGDG	956.631049
Lipid-B-P-1196	DGDG(18:3_18:3)	GL	DGDG	954.615399
Lipid-B-P-1203	DGDG(18:2_20:5)	GL	DGDG	980.631049
Lipid-B-P-1180	DGDG(18:1_18:3)	GL	DGDG	958.646699
Lipid-B-P-1165	DGDG(16:1_18:1)	GL	DGDG	934.646699

Lipid-B-P-1157	DGDG(16:0_18:1)	GL	DGDG	936.662349
Lipid-B-P-1158	DGDG(16:0_20:1)	GL	DGDG	964.693649
Lipid-B-P-1156	DGDG(14:0_18:1)	GL	DGDG	908.631049
Lipid-B-P-1154	DGDG(16:0_16:0)	GL	DGDG	910.646699
Lipid-B-P-1164	DGDG(16:0_18:2)	GL	DGDG	934.646699
Lipid-B-P-1044	DGTS(18:2_18:2)	GL	DGTS	760.6077
Lipid-B-P-1043	DGTS(18:1_18:3)	GL	DGTS	760.6089
Lipid-B-P-1036	DGTS(18:1_18:2)	GL	DGTS	762.6261
Lipid-B-P-1027	DGTS(18:1_18:1)	GL	DGTS	764.6437
Lipid-B-P-1025	DGTS(16:0_18:2)	GL	DGTS	736.6113
Lipid-B-P-1018	DGTS(16:0_18:1)	GL	DGTS	738.6224
Lipid-B-P-1012	DGTS(16:0_16:0)	GL	DGTS	712.6063
Lipid-B-P-1040	DGTS(14:0_18:4)	GL	DGTS	704.5442
Lipid-B-N-0001	FFA(10:0)	FA	FFA	171.138505
Lipid-B-N-0047	FFA(20:5)	FA	FFA	301.216755
Lipid-B-N-0008	FFA(17:0)	FA	FFA	269.248055
Lipid-B-N-0009	FFA(18:0)	FA	FFA	283.263705
Lipid-B-N-0012	FFA(22:0)	FA	FFA	339.326305
Lipid-B-N-0013	FFA(24:0)	FA	FFA	367.357605
Lipid-B-N-0015	FFA(28:0)	FA	FFA	423.42047
Lipid-B-N-0027	FFA(17:1)	FA	FFA	267.232405
Lipid-B-N-0028	FFA(18:1)	FA	FFA	281.248055
Lipid-B-N-0030	FFA(20:1)	FA	FFA	309.279355
Lipid-B-N-0031	FFA(22:1)	FA	FFA	337.310655
Lipid-B-N-0007	FFA(16:0)	FA	FFA	255.232405
Lipid-B-N-0039	FFA(18:3)	FA	FFA	277.216755
Lipid-B-N-0046	FFA(24:4)	FA	FFA	359.295005
Lipid-B-N-0051	FFA(24:6)	FA	FFA	355.263705
Lipid-B-N-0034	FFA(18:2)	FA	FFA	279.232405
Lipid-B-P-0160	HexCer(d18:1/24:1)	SP	HexCer	810.682325
Lipid-B-P-0159	HexCer(d18:1/22:1)	SP	HexCer	782.651025
Lipid-B-P-0158	HexCer(d18:1/20:1)	SP	HexCer	754.619725
Lipid-B-P-0157	HexCer(d18:1/18:1)	SP	HexCer	726.588425
Lipid-B-P-0156	HexCer(d18:1/16:1)	SP	HexCer	698.557125
Lipid-B-P-0146	HexCer(d18:1/16:0)	SP	HexCer	700.572744
Lipid-B-P-0139	HexCer(d16:1/18:0)	SP	HexCer	700.6
Lipid-B-P-0121	HexCer(t18:1/24:1(2OH))	SP	HexCer	842.672124
Lipid-B-P-0117	HexCer(t18:1/26:0(2OH))	SP	HexCer	872.719074
Lipid-B-P-0116	HexCer(t18:1/24:0(2OH))	SP	HexCer	844.687774
Lipid-B-P-0115	HexCer(t18:1/23:0(2OH))	SP	HexCer	830.6609
Lipid-B-P-0113	HexCer(t18:1/20:0(2OH))	SP	HexCer	788.625174
Lipid-B-P-0112	HexCer(t18:1/16:0(2OH))	SP	HexCer	732.562574
Lipid-B-P-0164	HexCer(d18:2/20:0)	SP	HexCer	754.6
Lipid-B-P-0165	HexCer(d18:2/22:0)	SP	HexCer	782.7
Lipid-B-P-0166	HexCer(d18:2/24:0)	SP	HexCer	810.7
Lipid-B-P-0162	HexCer(d18:2/16:0)	SP	HexCer	698.6
Lipid-B-P-0114	HexCer(t18:1/22:0(2OH))	SP	HexCer	816.656474
Lipid-B-P-0163	HexCer(d18:2/18:0)	SP	HexCer	726.6
Lipid-B-P-1105	LDGTS(18:2)	GL	LDGTS	498.377
Lipid-B-P-1107	LDGTS(18:3)	GL	LDGTS	496.3592
Lipid-B-P-1100	LDGTS(16:1)	GL	LDGTS	472.3628
Lipid-B-P-1096	LDGTS(18:0)	GL	LDGTS	502.4104
Lipid-B-P-1095	LDGTS(17:0)	GL	LDGTS	488.394
Lipid-B-P-1094	LDGTS(16:0)	GL	LDGTS	474.3782

Lipid-B-P-1102	LDGTS(18:1)	GL	LDGTS	500.3933
Lipid-B-N-0455	LPA(18:3)	GP	LPA	431.235517
Lipid-B-N-0459	LPA(22:5)	GP	LPA	483.235517
Lipid-B-N-0453	LPA(18:2)	GP	LPA	433.235517
Lipid-B-N-0448	LPA(16:0)	GP	LPA	409.235515
Lipid-B-N-0449	LPA(18:0)	GP	LPA	437.266815
Lipid-B-N-0451	LPA(18:1)	GP	LPA	435.258993
Lipid-B-P-0200	LPC(22:6)	GP	LPC	568.340317
Lipid-B-P-0189	LPC(18:2)	GP	LPC	520.340317
Lipid-B-P-0188	LPC(16:2)	GP	LPC	494.324667
Lipid-B-P-0185	LPC(20:1)	GP	LPC	550.387267
Lipid-B-P-0184	LPC(19:1)	GP	LPC	536.37085
Lipid-B-P-0193	LPC(18:3)	GP	LPC	518.324667
Lipid-B-P-0182	LPC(17:1)	GP	LPC	508.33932
Lipid-B-P-0181	LPC(16:1)	GP	LPC	494.324667
Lipid-B-P-0180	LPC(15:1)	GP	LPC	480.30682
Lipid-B-P-0176	LPC(22:0)	GP	LPC	580.434217
Lipid-B-P-0169	LPC(14:0)	GP	LPC	468.309017
Lipid-B-P-0175	LPC(20:0)	GP	LPC	552.402917
Lipid-B-P-0171	LPC(16:0)	GP	LPC	496.340317
Lipid-B-P-0172	LPC(17:0)	GP	LPC	510.35565
Lipid-B-P-0173	LPC(18:0)	GP	LPC	524.371617
Lipid-B-P-0183	LPC(18:1)	GP	LPC	522.355967
Lipid-B-P-0218	LPE(18:3)	GP	LPE	476.277714
Lipid-B-P-0214	LPE(18:2)	GP	LPE	478.293364
Lipid-B-P-0207	LPE(16:1)	GP	LPE	452.277714
Lipid-B-P-0208	LPE(17:1)	GP	LPE	466.29028
Lipid-B-P-0206	LPE(24:0)	GP	LPE	566.418565
Lipid-B-P-0205	LPE(22:0)	GP	LPE	538.387265
Lipid-B-P-0204	LPE(20:0)	GP	LPE	510.355965
Lipid-B-P-0201	LPE(16:0)	GP	LPE	454.293364
Lipid-B-P-0209	LPE(18:1)	GP	LPE	480.309014
Lipid-B-N-0056	LPG(18:1)	GP	LPG	509.287947
Lipid-B-N-0052	LPG(16:0)	GP	LPG	483.272297
Lipid-B-N-0053	LPG(18:0)	GP	LPG	511.303597
Lipid-B-N-0058	LPG(18:2)	GP	LPG	507.272297
Lipid-B-N-0066	LPI(16:0)	GP	LPI	571.288343
Lipid-B-N-0069	LPI(18:1)	GP	LPI	597.303993
Lipid-B-N-0071	LPI(18:2)	GP	LPI	595.288343
Lipid-B-P-0457	MG(16:0)	GL	MG	348.320751
Lipid-B-P-0458	MG(18:0)	GL	MG	376.352051
Lipid-B-P-0463	MG(18:1)	GL	MG	374.336401
Lipid-B-P-0465	MG(18:2)	GL	MG	372.31012
Lipid-B-P-0468	MG(18:3)	GL	MG	370.31012
Lipid-B-P-1235	MGDG(18:1_18:2)	GL	MGDG	825.572805
Lipid-B-P-1232	MGDG(16:0_18:3)	GL	MGDG	797.541505
Lipid-B-P-1224	MGDG(16:1_18:1)	GL	MGDG	799.557155
Lipid-B-P-1246	MGDG(18:1_18:3)	GL	MGDG	823.557155
Lipid-B-P-1269	MGDG(18:3_18:3)	GL	MGDG	819.525855
Lipid-B-P-1226	MGDG(18:1_18:1)	GL	MGDG	827.588455
Lipid-B-P-1260	MGDG(18:2_18:3)	GL	MGDG	821.541505
Lipid-B-P-1223	MGDG(16:0_18:2)	GL	MGDG	799.557155
Lipid-B-P-1217	MGDG(16:0_18:1)	GL	MGDG	801.572805
Lipid-B-P-1212	MGDG(14:0_16:0)	GL	MGDG	747.525855

Lipid-B-P-1247	MGDG(18:2_18:2)	GL	MGDG	823.557155
Lipid-B-P-1213	MGDG(16:0_16:0)	GL	MGDG	775.557155
Lipid-B-P-1275	MGDG(18:2_20:5)	GL	MGDG	845.541505
Lipid-B-P-1227	MGDG(18:1_19:1)	GL	MGDG	841.604105
Lipid-B-N-0463	PA(16:0_16:0)	GP	PA	647.465182
Lipid-B-N-0467	PA(16:0_16:1)	GP	PA	645.449532
Lipid-B-N-0474	PA(16:0_18:2)	GP	PA	671.465182
Lipid-B-N-0475	PA(18:0_18:2)	GP	PA	699.496482
Lipid-B-N-0476	PA(18:2_20:0)	GP	PA	727.527782
Lipid-B-N-0479	PA(18:3_16:0)	GP	PA	669.449532
Lipid-B-N-0480	PA(18:2_18:1)	GP	PA	697.480832
Lipid-B-N-0485	PA(18:1_18:3)	GP	PA	695.465182
Lipid-B-N-0489	PA(18:2_18:3)	GP	PA	693.449532
Lipid-B-N-0462	PA(16:0_15:0)	GP	PA	633.449532
Lipid-B-N-0465	PA(18:0_18:0)	GP	PA	703.527782
Lipid-B-N-0468	PA(16:0_18:1)	GP	PA	673.480832
Lipid-B-N-0113	PC(14:0_18:1)	GP	PC	776.544161
Lipid-B-N-0105	PC(18:0_14:0)	GP	PC	778.559811
Lipid-B-N-0115	PC(16:0_18:1)	GP	PC	804.575461
Lipid-B-N-0201	PE(16:1_16:0)	GP	PE	688.491732
Lipid-B-N-0220	PE(18:1_18:1)	GP	PE	742.538683
Lipid-B-N-0195	PE(16:0_16:0)	GP	PE	690.507383
Lipid-B-N-0194	PE(16:0_14:0)	GP	PE	662.476082
Lipid-B-N-0203	PE(16:0_18:1)	GP	PE	716.523033
Lipid-B-N-0218	PE(18:2_16:0)	GP	PE	714.507383
Lipid-B-N-0291	PG(18:0_16:0)	GP	PG	749.541089
Lipid-B-N-0294	PG(18:1_14:0)	GP	PG	719.494139
Lipid-B-N-0296	PG(16:0_18:1)	GP	PG	747.525439
Lipid-B-N-0299	PG(18:0_18:1)	GP	PG	775.556739
Lipid-B-N-0304	PG(18:2_16:0)	GP	PG	745.509789
Lipid-B-N-0306	PG(18:1_18:1)	GP	PG	773.541089
Lipid-B-N-0309	PG(20:1_18:1)	GP	PG	801.572389
Lipid-B-N-0289	PG(16:0_16:0)	GP	PG	721.509789
Lipid-B-N-0384	PI(18:1_18:3)	GP	PI	857.525834
Lipid-B-N-0382	PI(18:2_18:2)	GP	PI	857.525834
Lipid-B-N-0374	PI(18:1_18:2)	GP	PI	859.541484
Lipid-B-N-0372	PI(18:3_16:0)	GP	PI	831.510184
Lipid-B-N-0371	PI(18:2_16:1)	GP	PI	831.510184
Lipid-B-N-0364	PI(18:1_18:1)	GP	PI	861.557134
Lipid-B-N-0363	PI(18:0_18:2)	GP	PI	861.557134
Lipid-B-N-0361	PI(17:1_18:1)	GP	PI	847.533658
Lipid-B-N-0351	PI(18:0_18:1)	GP	PI	863.572784
Lipid-B-N-0348	PI(16:0_18:1)	GP	PI	835.541484
Lipid-B-N-0342	PI(16:0_20:0)	GP	PI	865.588434
Lipid-B-N-0341	PI(16:0_18:0)	GP	PI	837.557134
Lipid-B-N-0340	PI(16:0_16:0)	GP	PI	809.518007
Lipid-B-N-0359	PI(18:2_16:0)	GP	PI	833.525834
Lipid-B-N-0523	PMeOH(20:2_22:5)	GP	PMeOH	787.5245
Lipid-B-N-0522	PMeOH(20:2_20:5)	GP	PMeOH	759.4929
Lipid-B-N-0506	PMeOH(14:0_16:0)	GP	PMeOH	633.4456
Lipid-B-N-0427	PS(18:0_18:2)	GP	PS	786.528511
Lipid-B-N-0429	PS(18:1_20:1)	GP	PS	814.559811
Lipid-B-N-0420	PS(18:1_19:0)	GP	PS	802.559811
Lipid-B-P-0008	SPH(d18:2)	SP	SPH	298.274604

Lipid-B-P-0007	SPH(d18:1)	SP	SPH	300.290254
Lipid-B-P-0006	SPH(d16:1)	SP	SPH	272.258954
Lipid-B-P-0005	SPH(d18:0)	SP	SPH	302.305904
Lipid-B-P-0003	PhytoSph(d16:2)	SP	SPH	286.238219
Lipid-B-P-0002	PhytoSph(d18:1)	SP	SPH	316.285169
Lipid-B-P-0001	PhytoSph(d18:0)	SP	SPH	318.300819
Lipid-B-P-1116	SQDG(16:0_16:0)	GL	SQDG	812.55731
Lipid-B-P-1141	SQDG(18:2_18:2)	GL	SQDG	860.5555
Lipid-B-P-1140	SQDG(18:1_18:3)	GL	SQDG	860.5509
Lipid-B-P-1115	SQDG(14:0_16:0)	GL	SQDG	784.5226
Lipid-B-P-1135	SQDG(16:0_18:3)	GL	SQDG	834.54059
Lipid-B-P-1129	SQDG(18:1_18:1)	GL	SQDG	864.5858
Lipid-B-P-1128	SQDG(16:0_18:2)	GL	SQDG	836.55658
Lipid-B-P-1122	SQDG(16:0_18:1)	GL	SQDG	838.5705
Lipid-B-P-1121	SQDG(16:0_17:1)	GL	SQDG	824.556
Lipid-B-P-1120	SQDG(16:0_16:1)	GL	SQDG	810.5419
Lipid-B-P-1136	SQDG(18:1_18:2)	GL	SQDG	862.5653
Lipid-B-P-0734	TG(20:0_18:2_18:2)	GL	TG	928.833314
Lipid-B-P-0735	TG(18:2_21:0_18:2)	GL	TG	942.848964
Lipid-B-P-0736	TG(18:1_22:1_18:2)	GL	TG	956.864614
Lipid-B-P-0738	TG(18:1_18:3_22:0)	GL	TG	956.864614
Lipid-B-P-0739	TG(18:2_18:2_22:0)	GL	TG	956.864614
Lipid-B-P-0740	TG(20:1_20:1_18:2)	GL	TG	956.864614
Lipid-B-P-0741	TG(18:3_20:0_20:1)	GL	TG	956.864614
Lipid-B-P-0744	TG(18:1_20:1_20:2)	GL	TG	956.864614
Lipid-B-P-0745	TG(17:1_18:1_24:2)	GL	TG	970.880264
Lipid-B-P-0751	TG(18:3_20:0_22:1)	GL	TG	984.895914
Lipid-B-P-0749	TG(18:1_20:2_22:1)	GL	TG	984.895914
Lipid-B-P-0753	TG(18:1_24:0_18:3)	GL	TG	984.895914
Lipid-B-P-0754	TG(18:2_24:0_18:2)	GL	TG	984.895914
Lipid-B-P-0755	TG(25:0_18:2_18:2)	GL	TG	998.911564
Lipid-B-P-0756	TG(20:1_24:1_18:2)	GL	TG	1012.927214
Lipid-B-P-0733	TG(18:1_18:1_20:2)	GL	TG	928.833314
Lipid-B-P-0766	TG(14:0_18:2_18:3)	GL	TG	842.723764
Lipid-B-P-0770	TG(14:0_14:0_22:5)	GL	TG	842.723764
Lipid-B-P-0775	TG(15:0_18:2_18:3)	GL	TG	856.739414
Lipid-B-P-0776	TG(15:1_18:2_18:2)	GL	TG	856.739414
Lipid-B-P-0746	TG(23:0_18:2_18:2)	GL	TG	970.880264
Lipid-B-P-0731	TG(18:0_18:3_20:1)	GL	TG	928.833314
Lipid-B-P-0723	TG(16:0_18:2_20:2)	GL	TG	900.802014
Lipid-B-P-0729	TG(18:1_18:3_20:0)	GL	TG	928.833314
Lipid-B-P-0687	TG(18:1_18:2_26:0)	GL	TG	1014.942864
Lipid-B-P-0701	TG(15:1_17:1_17:2)	GL	TG	830.723764
Lipid-B-P-0702	TG(13:0_18:1_18:3)	GL	TG	830.723764
Lipid-B-P-0703	TG(13:0_16:0_20:4)	GL	TG	830.723764
Lipid-B-P-0704	TG(14:0_18:2_18:2)	GL	TG	844.739414
Lipid-B-P-0705	TG(16:1_16:1_18:2)	GL	TG	844.739414
Lipid-B-P-0706	TG(16:0_16:1_18:3)	GL	TG	844.739414
Lipid-B-P-0708	TG(14:0_18:1_18:3)	GL	TG	844.739414
Lipid-B-P-0712	TG(16:0_16:2_18:2)	GL	TG	844.739414
Lipid-B-P-0713	TG(15:0_18:2_18:2)	GL	TG	858.755064
Lipid-B-P-0730	TG(18:0_18:2_20:2)	GL	TG	928.833314
Lipid-B-P-0714	TG(16:0_17:1_18:3)	GL	TG	858.755064
Lipid-B-P-0717	TG(16:0_18:1_18:3)	GL	TG	872.770714

Lipid-B-P-0718	TG(17:1_18:1_18:2)	GL	TG	886.786364
Lipid-B-P-0720	TG(17:0_18:2_18:2)	GL	TG	886.786364
Lipid-B-P-0721	TG(18:1_18:1_18:2)	GL	TG	900.802014
Lipid-B-P-0722	TG(16:0_18:3_20:1)	GL	TG	900.802014
Lipid-B-P-0724	TG(16:0_18:1_20:3)	GL	TG	900.802014
Lipid-B-P-0725	TG(18:0_18:1_18:3)	GL	TG	900.802014
Lipid-B-P-0726	TG(18:1_19:1_18:2)	GL	TG	914.817664
Lipid-B-P-0727	TG(16:0_18:3_22:1)	GL	TG	928.833314
Lipid-B-P-0728	TG(16:0_20:2_20:2)	GL	TG	928.833314
Lipid-B-P-0716	TG(16:0_18:2_18:2)	GL	TG	872.770714
Lipid-B-P-0777	TG(13:0_16:0_22:5)	GL	TG	856.739414
Lipid-B-P-0787	TG(17:1_18:1_18:3)	GL	TG	884.770714
Lipid-B-P-0780	TG(16:0_18:2_18:3)	GL	TG	870.755064
Lipid-B-P-0833	TG(18:1_18:2_18:3)	GL	TG	896.770714
Lipid-B-P-0835	TG(18:2_18:2_18:2)	GL	TG	896.770714
Lipid-B-P-0837	TG(18:1_18:1_18:4)	GL	TG	896.770714
Lipid-B-P-0838	TG(18:0_18:3_18:3)	GL	TG	896.770714
Lipid-B-P-0841	TG(18:2_18:2_20:2)	GL	TG	924.802014
Lipid-B-P-0842	TG(18:3_18:3_20:0)	GL	TG	924.802014
Lipid-B-P-0844	TG(18:2_18:3_20:1)	GL	TG	924.802014
Lipid-B-P-0861	TG(16:0_18:2_18:5)	GL	TG	866.723764
Lipid-B-P-0863	TG(12:0_18:1_22:6)	GL	TG	866.723764
Lipid-B-P-0869	TG(18:2_18:2_18:3)	GL	TG	894.755064
Lipid-B-P-0870	TG(18:1_18:2_18:4)	GL	TG	894.755064
Lipid-B-P-0875	TG(18:3_18:3_20:1)	GL	TG	922.786364
Lipid-B-P-0897	TG(18:2_18:3_18:3)	GL	TG	892.739414
Lipid-B-P-0899	TG(16:0_18:3_20:5)	GL	TG	892.739414
Lipid-B-P-0901	TG(18:1_18:2_20:5)	GL	TG	920.770714
Lipid-B-P-0914	TG(18:3_18:3_18:3)	GL	TG	890.723764
Lipid-B-P-0918	TG(16:0_20:4_22:5)	GL	TG	946.786364
Lipid-B-P-0929	TG(18:2_18:4_18:4)	GL	TG	888.708114
Lipid-B-P-0931	TG(16:3_18:2_22:5)	GL	TG	916.739414
Lipid-B-P-0934	TG(16:0_20:5_20:5)	GL	TG	916.739414
Lipid-B-P-0685	TG(18:2_20:1_22:0)	GL	TG	986.911564
Lipid-B-P-0832	TG(17:0_18:3_18:3)	GL	TG	882.755064
Lipid-B-P-0829	TG(16:0_18:3_18:3)	GL	TG	868.739414
Lipid-B-P-0828	TG(16:1_16:1_20:4)	GL	TG	868.739414
Lipid-B-P-0825	TG(16:0_18:2_18:4)	GL	TG	868.739414
Lipid-B-P-0781	TG(14:0_16:0_22:5)	GL	TG	870.755064
Lipid-B-P-0782	TG(16:3_18:1_18:1)	GL	TG	870.755064
Lipid-B-P-0784	TG(16:0_18:1_18:4)	GL	TG	870.755064
Lipid-B-P-0786	TG(17:1_18:2_18:2)	GL	TG	884.770714
Lipid-B-P-0789	TG(17:0_18:2_18:3)	GL	TG	884.770714
Lipid-B-P-0790	TG(18:1_18:2_18:2)	GL	TG	898.786364
Lipid-B-P-0791	TG(16:0_18:1_20:4)	GL	TG	898.786364
Lipid-B-P-0792	TG(18:0_18:2_18:3)	GL	TG	898.786364
Lipid-B-P-0793	TG(16:0_18:3_20:2)	GL	TG	898.786364
Lipid-B-P-0795	TG(18:1_18:1_18:3)	GL	TG	898.786364
Lipid-B-P-0779	TG(16:1_18:2_18:2)	GL	TG	870.755064
Lipid-B-P-0798	TG(18:1_18:2_20:2)	GL	TG	926.817664
Lipid-B-P-0800	TG(18:2_18:2_20:1)	GL	TG	926.817664
Lipid-B-P-0801	TG(18:2_18:3_20:0)	GL	TG	926.817664
Lipid-B-P-0802	TG(18:1_18:3_20:1)	GL	TG	926.817664
Lipid-B-P-0804	TG(18:2_18:2_22:1)	GL	TG	954.848964

Lipid-B-P-0806	TG(18:2_18:3_22:0)	GL	TG	954.848964
Lipid-B-P-0807	TG(18:1_18:3_22:1)	GL	TG	954.848964
Lipid-B-P-0809	TG(18:3_20:1_20:1)	GL	TG	954.848964
Lipid-B-P-0813	TG(24:0_18:2_18:3)	GL	TG	982.880264
Lipid-B-P-0823	TG(15:0_18:3_18:3)	GL	TG	854.723764
Lipid-B-P-0824	TG(16:0_16:1_20:5)	GL	TG	868.739414
Lipid-B-P-0799	TG(18:0_18:3_20:2)	GL	TG	926.817664
Lipid-B-P-0683	TG(18:1_22:0_20:2)	GL	TG	986.911564
Lipid-B-P-0609	TG(16:0_23:0_18:2)	GL	TG	946.880264
Lipid-B-P-0681	TG(23:0_18:1_18:2)	GL	TG	972.895914
Lipid-B-P-0541	TG(18:0_18:0_18:1)	GL	TG	906.848964
Lipid-B-P-0540	TG(16:0_18:0_20:1)	GL	TG	906.848964
Lipid-B-P-0539	TG(17:0_18:0_18:1)	GL	TG	892.833314
Lipid-B-P-0538	TG(16:0_16:0_20:1)	GL	TG	878.817664
Lipid-B-P-0537	TG(16:0_16:1_20:0)	GL	TG	878.817664
Lipid-B-P-0536	TG(16:0_18:0_18:1)	GL	TG	878.817664
Lipid-B-P-0535	TG(16:0_17:0_18:1)	GL	TG	864.802014
Lipid-B-P-0542	TG(16:0_20:0_18:1)	GL	TG	906.848964
Lipid-B-P-0534	TG(16:0_16:0_18:1)	GL	TG	850.786364
Lipid-B-P-0531	TG(15:0_16:0_18:1)	GL	TG	836.770714
Lipid-B-P-0530	TG(14:0_16:0_18:1)	GL	TG	822.755064
Lipid-B-P-0529	TG(16:0_16:0_16:1)	GL	TG	822.755064
Lipid-B-P-0528	TG(16:0_16:0_15:1)	GL	TG	808.739414
Lipid-B-P-0527	TG(13:0_16:0_18:1)	GL	TG	808.739414
Lipid-B-P-0526	TG(15:0_16:0_16:1)	GL	TG	808.739414
Lipid-B-P-0525	TG(14:0_14:0_18:1)	GL	TG	794.723764
Lipid-B-P-0533	TG(16:0_16:1_17:0)	GL	TG	836.770714
Lipid-B-P-0524	TG(14:1_16:0_16:0)	GL	TG	794.723764
Lipid-B-P-0544	TG(16:0_18:1_20:0)	GL	TG	906.848964
Lipid-B-P-0546	TG(16:0_18:1_21:0)	GL	TG	920.864614
Lipid-B-P-0573	TG(14:1_16:0_18:1)	GL	TG	820.739414
Lipid-B-P-0572	TG(14:0_16:1_18:1)	GL	TG	820.739414
Lipid-B-P-0571	TG(14:0_16:0_18:2)	GL	TG	820.739414
Lipid-B-P-0570	TG(16:0_16:1_16:1)	GL	TG	820.739414
Lipid-B-P-0569	TG(15:0_16:1_16:1)	GL	TG	806.723764
Lipid-B-P-0566	TG(14:1_16:0_16:1)	GL	TG	792.708114
Lipid-B-P-0558	TG(16:0_26:0_18:1)	GL	TG	990.942864
Lipid-B-P-0545	TG(15:0_16:0_24:1)	GL	TG	920.864614
Lipid-B-P-0557	TG(16:0_25:0_18:1)	GL	TG	976.927214
Lipid-B-P-0555	TG(16:0_24:0_18:1)	GL	TG	962.911564
Lipid-B-P-0554	TG(16:0_23:0_18:1)	GL	TG	948.895914
Lipid-B-P-0553	TG(15:0_24:0_18:1)	GL	TG	948.895914
Lipid-B-P-0551	TG(16:0_20:0_20:1)	GL	TG	934.880264
Lipid-B-P-0549	TG(14:0_20:1_22:0)	GL	TG	934.880264
Lipid-B-P-0548	TG(16:0_18:1_22:0)	GL	TG	934.880264
Lipid-B-P-0547	TG(18:0_18:1_20:0)	GL	TG	934.880264
Lipid-B-P-0556	TG(16:0_20:1_22:0)	GL	TG	962.911564
Lipid-B-P-0575	TG(16:0_16:1_17:1)	GL	TG	834.755064
Lipid-B-P-0523	TG(14:0_16:0_16:1)	GL	TG	794.723764
Lipid-B-P-0521	TG(13:0_14:0_18:1)	GL	TG	780.708114
Lipid-B-P-0494	TG(16:0_17:0_18:0)	GL	TG	866.817664
Lipid-B-P-0493	TG(15:0_17:0_18:0)	GL	TG	852.802014
Lipid-B-P-0492	TG(16:0_16:0_18:0)	GL	TG	852.802014
Lipid-B-P-0491	TG(15:0_16:0_18:0)	GL	TG	838.786364

Lipid-B-P-0490	TG(16:0_16:0_17:0)	GL	TG	838.786364
Lipid-B-P-0489	TG(15:0_16:0_17:0)	GL	TG	824.770714
Lipid-B-P-0488	TG(14:0_16:0_18:0)	GL	TG	824.770714
Lipid-B-P-0495	TG(16:0_18:0_18:0)	GL	TG	880.833316
Lipid-B-P-0487	TG(16:0_16:0_16:0)	GL	TG	824.770714
Lipid-B-P-0485	TG(15:0_16:0_16:0)	GL	TG	810.755064
Lipid-B-P-0484	TG(14:0_16:0_16:0)	GL	TG	796.739416
Lipid-B-P-0483	TG(14:0_15:0_16:0)	GL	TG	782.723764
Lipid-B-P-0482	TG(13:0_15:0_16:0)	GL	TG	768.708114
Lipid-B-P-0480	TG(14:0_14:0_16:0)	GL	TG	768.708114
Lipid-B-P-0479	TG(13:0_14:0_16:0)	GL	TG	754.692464
Lipid-B-P-0478	TG(12:0_14:0_16:0)	GL	TG	740.676814
Lipid-B-P-0486	TG(14:0_16:0_17:0)	GL	TG	810.755064
Lipid-B-P-0522	TG(12:0_16:0_18:1)	GL	TG	794.723764
Lipid-B-P-0496	TG(16:0_16:0_20:0)	GL	TG	880.833314
Lipid-B-P-0499	TG(17:0_17:0_19:0)	GL	TG	894.848964
Lipid-B-P-0520	TG(14:0_15:0_16:1)	GL	TG	780.708114
Lipid-B-P-0519	TG(14:0_14:0_16:1)	GL	TG	766.692464
Lipid-B-P-0518	TG(12:0_14:0_18:1)	GL	TG	766.692464
Lipid-B-P-0517	TG(12:0_16:0_16:1)	GL	TG	766.692464
Lipid-B-P-0515	TG(12:0_14:0_16:1)	GL	TG	738.661164
Lipid-B-P-0510	TG(16:0_16:0_25:0)	GL	TG	950.911564
Lipid-B-P-0509	TG(16:0_18:0_23:0)	GL	TG	950.911564
Lipid-B-P-0497	TG(15:0_18:0_20:0)	GL	TG	894.848964
Lipid-B-P-0508	TG(16:0_20:0_20:0)	GL	TG	936.895914
Lipid-B-P-0506	TG(16:0_18:0_22:0)	GL	TG	936.895914
Lipid-B-P-0505	TG(16:0_16:0_23:0)	GL	TG	922.880264
Lipid-B-P-0504	TG(15:0_16:0_24:0)	GL	TG	922.880264
Lipid-B-P-0503	TG(16:0_16:0_22:0)	GL	TG	908.864614
Lipid-B-P-0502	TG(14:0_18:0_22:0)	GL	TG	908.864614
Lipid-B-P-0501	TG(16:0_18:0_20:0)	GL	TG	908.864614
Lipid-B-P-0500	TG(18:0_18:0_18:0)	GL	TG	908.864614
Lipid-B-P-0507	TG(16:0_16:0_24:0)	GL	TG	936.895914
Lipid-B-P-0682	TG(24:0_18:1_18:2)	GL	TG	986.911564
Lipid-B-P-0576	TG(15:0_16:0_18:2)	GL	TG	834.755064
Lipid-B-P-0578	TG(14:0_17:1_18:1)	GL	TG	834.755064
Lipid-B-P-0657	TG(18:1_18:1_18:1)	GL	TG	902.817664
Lipid-B-P-0655	TG(17:0_18:1_18:2)	GL	TG	888.802014
Lipid-B-P-0654	TG(16:0_18:0_18:3)	GL	TG	874.786364
Lipid-B-P-0653	TG(14:0_18:1_20:2)	GL	TG	874.786364
Lipid-B-P-0652	TG(16:1_18:1_18:1)	GL	TG	874.786364
Lipid-B-P-0651	TG(16:0_18:1_18:2)	GL	TG	874.786364
Lipid-B-P-0650	TG(16:0_17:0_18:3)	GL	TG	860.770714
Lipid-B-P-0658	TG(18:0_18:1_18:2)	GL	TG	902.817664
Lipid-B-P-0647	TG(16:0_17:1_18:2)	GL	TG	860.770714
Lipid-B-P-0645	TG(14:0_18:1_18:2)	GL	TG	846.755064
Lipid-B-P-0644	TG(16:0_16:0_18:3)	GL	TG	846.755064
Lipid-B-P-0643	TG(16:0_16:2_18:1)	GL	TG	846.755064
Lipid-B-P-0642	TG(16:0_16:1_18:2)	GL	TG	846.755064
Lipid-B-P-0641	TG(16:1_16:1_18:1)	GL	TG	846.755064
Lipid-B-P-0640	TG(16:0_15:1_18:2)	GL	TG	832.739414
Lipid-B-P-0636	TG(14:0_16:0_18:3)	GL	TG	818.723764
Lipid-B-P-0646	TG(15:0_18:1_18:2)	GL	TG	860.770714
Lipid-B-P-0635	TG(14:0_16:1_18:2)	GL	TG	818.723764

Lipid-B-P-0659	TG(16:0_18:1_20:2)	GL	TG	902.817664
Lipid-B-P-0661	TG(16:0_18:2_20:1)	GL	TG	902.817664
Lipid-B-P-0678	TG(18:3_20:0_20:0)	GL	TG	958.880264
Lipid-B-P-0677	TG(18:2_20:0_20:1)	GL	TG	958.880264
Lipid-B-P-0675	TG(18:1_18:1_22:1)	GL	TG	958.880264
Lipid-B-P-0674	TG(18:1_18:2_22:0)	GL	TG	958.880264
Lipid-B-P-0673	TG(21:0_18:1_18:2)	GL	TG	944.864614
Lipid-B-P-0672	TG(18:0_18:3_20:0)	GL	TG	930.848964
Lipid-B-P-0671	TG(16:0_18:2_22:1)	GL	TG	930.848964
Lipid-B-P-0660	TG(18:0_18:0_18:3)	GL	TG	902.817664
Lipid-B-P-0670	TG(16:0_20:1_20:2)	GL	TG	930.848964
Lipid-B-P-0668	TG(20:1_18:2_18:0)	GL	TG	930.848964
Lipid-B-P-0667	TG(18:1_18:1_20:1)	GL	TG	930.848964
Lipid-B-P-0666	TG(18:1_18:2_20:0)	GL	TG	930.848964
Lipid-B-P-0665	TG(18:1_18:1_19:1)	GL	TG	916.833314
Lipid-B-P-0664	TG(17:1_18:1_20:1)	GL	TG	916.833314
Lipid-B-P-0663	TG(16:0_18:3_20:0)	GL	TG	902.817664
Lipid-B-P-0662	TG(20:0_18:1_18:2)	GL	TG	902.817664
Lipid-B-P-0669	TG(16:0_18:3_22:0)	GL	TG	930.848964
Lipid-B-P-0577	TG(13:0_18:1_18:1)	GL	TG	834.755064
Lipid-B-P-0622	TG(26:0_18:1_18:1)	GL	TG	1016.958514
Lipid-B-P-0620	TG(18:0_20:1_22:1)	GL	TG	988.927214
Lipid-B-P-0597	TG(18:0_18:0_18:2)	GL	TG	904.833314
Lipid-B-P-0595	TG(16:0_18:2_20:0)	GL	TG	904.833314
Lipid-B-P-0593	TG(16:1_18:0_20:1)	GL	TG	904.833314
Lipid-B-P-0592	TG(16:0_18:1_20:1)	GL	TG	904.833314
Lipid-B-P-0591	TG(18:0_18:1_18:1)	GL	TG	904.833314
Lipid-B-P-0590	TG(17:0_18:0_18:2)	GL	TG	890.817664
Lipid-B-P-0589	TG(17:0_18:1_18:1)	GL	TG	890.817664
Lipid-B-P-0598	TG(15:0_18:1_22:1)	GL	TG	918.848964
Lipid-B-P-0588	TG(16:0_16:0_20:2)	GL	TG	876.802014
Lipid-B-P-0586	TG(16:0_18:1_18:1)	GL	TG	876.802014
Lipid-B-P-0585	TG(16:0_17:0_18:2)	GL	TG	862.786364
Lipid-B-P-0584	TG(16:0_17:1_18:1)	GL	TG	862.786364
Lipid-B-P-0583	TG(16:0_16:0_18:2)	GL	TG	848.770714
Lipid-B-P-0582	TG(14:0_16:1_20:1)	GL	TG	848.770714
Lipid-B-P-0581	TG(16:0_16:1_18:1)	GL	TG	848.770714
Lipid-B-P-0580	TG(16:1_16:1_17:0)	GL	TG	834.755064
Lipid-B-P-0587	TG(16:0_18:0_18:2)	GL	TG	876.802014
Lipid-B-P-0621	TG(24:0_18:1_20:1)	GL	TG	1016.958514
Lipid-B-P-0599	TG(18:0_18:1_19:1)	GL	TG	918.848964
Lipid-B-P-0601	TG(18:0_18:1_20:1)	GL	TG	932.864614
Lipid-B-P-0619	TG(24:0_18:1_18:1)	GL	TG	988.927214
Lipid-B-P-0618	TG(23:0_18:1_18:1)	GL	TG	974.911564
Lipid-B-P-0617	TG(18:1_20:0_22:1)	GL	TG	960.895914
Lipid-B-P-0616	TG(18:0_20:0_20:2)	GL	TG	960.895914
Lipid-B-P-0615	TG(18:2_20:0_20:0)	GL	TG	960.895914
Lipid-B-P-0614	TG(18:1_20:0_20:1)	GL	TG	960.895914
Lipid-B-P-0613	TG(16:0_24:0_18:2)	GL	TG	960.895914
Lipid-B-P-0600	TG(16:0_21:0_18:2)	GL	TG	918.848964
Lipid-B-P-0612	TG(16:0_18:1_24:1)	GL	TG	960.895914
Lipid-B-P-0610	TG(22:0_18:1_18:1)	GL	TG	960.895914
Lipid-B-P-0608	TG(15:0_18:1_24:1)	GL	TG	946.880264
Lipid-B-P-0607	TG(18:0_18:0_20:2)	GL	TG	932.864614

Lipid-B-P-0606	TG(18:1_18:1_20:0)	GL	TG	932.864614
Lipid-B-P-0604	TG(16:0_18:2_22:0)	GL	TG	932.864614
Lipid-B-P-0603	TG(16:0_18:1_22:1)	GL	TG	932.864614
Lipid-B-P-0602	TG(18:0_18:2_20:0)	GL	TG	932.864614
Lipid-B-P-0611	TG(18:0_22:0_18:2)	GL	TG	960.895914
Lipid-B-P-0532	TG(16:0_16:0_17:1)	GL	TG	836.770714

Molecular Weight	Ionization model	cpd_ID	lipidmaps ID	Pubchem CID
1032.78408	[M+NH4]+	-	-	-
1006.76843	[M+NH4]+	-	-	-
1008.78408	[M+NH4]+	-	-	-
651.652944	[M+H]+	C00195	LMSP02020012	5283577
563.527744	[M+H]+	C00195	LMSP02010003	5283563
535.496444	[M+H]+	C00195	-	131751776
677.668594	[M+H]+	C00195	LMSP02010011	5283570
649.637294	[M+H]+	C00195	LMSP02010012	5283571
635.621644	[M+H]+	C00195	LMSP02010021	52931115
621.605994	[M+H]+	C00195	LMSP02010008	5283567
607.590344	[M+H]+	C00195	-	-
623.621644	[M+H]+	C00195	LMSP02020010	5283575
595.590344	[M+H]+	C00195	LMSP02020009	5283574
567.559044	[M+H]+	C00195	LMSP02020008	5283573
539.527744	[M+H]+	C00195	LMSP02020001	5283572
667.647859	[M+H]+	C00195	LMSP02020033	53262287
591.559044	[M+H]+	C00195	-	-
619.590344	[M+H]+	C00195	-	53481052
663.652944	[M+H]+	C00195	LMSP02010013	9547202
675.652944	[M+H]+	C00195	LMSP02010010	5283569
669.605994	[M+H]+	C00195	-	-
557.480794	[M+H]+	C00195	-	-
647.621644	[M+H]+	C00195	LMSP02010009	5283568
645.605994	[M+H]+	C00195	-	-
617.574694	[M+H]+	C00195	-	-
589.543394	[M+H]+	C00195	LMSP02010027	52931121
561.512094	[M+H]+	C00195	LMSP02010025	52931119
559.496444	[M+H]+	C00195	-	-
675.652944	[M+H]+	C00195	-	-
647.621644	[M+H]+	C00195	-	-
633.605994	[M+H]+	C00195	LMSP02010030	52931124
619.590344	[M+H]+	C00195	LMSP02010029	52931123
535.496444	[M+H]+	C00195	LMSP02010024	52931118
533.480794	[M+H]+	C00195	-	-
667.611474	[M+H]+	C00195	LMSP02010186	-
695.642774	[M+H]+	C00195	LMSP02010184	-
709.658424	[M+H]+	C00195	LMSP02010179	-
723.674074	[M+H]+	C00195	LMSP02010180	-
653.632209	[M+H]+	C00195	-	-
639.616559	[M+H]+	C00195	LMSP02030008	5322153
583.553959	[M+H]+	C00195	LMSP02030006	9898642
693.663509	[M+H]+	C00195	LMSP02010138	-
599.548874	[M+H]+	C00195	LMSP02030016	44625889
667.647859	[M+H]+	C00195	LMSP02030004	10462091
653.595824	[M+H]+	C00195	LMSP02010187	-
555.522659	[M+H]+	C00195	LMSP02030001	10506988
625.564524	[M+H]+	C00195	LMSP02010189	-
627.580174	[M+H]+	C00195	LMSP02030017	70678864
569.501924	[M+H]+	C00195	LMSP02010193	-
711.674074	[M+H]+	C00195	LMSP02030023	70678890
709.658424	[M+H]+	C00195	-	-
681.627124	[M+H]+	C00195	-	-
711.674074	[M+H]+	C00195	LMSP02030003	52931128

697.658424	[M+H]+	C00195	LMSP02030068	-
683.642774	[M+H]+	C00195	LMSP02030002	52931127
669.627124	[M+H]+	C00195	LMSP02030067	-
655.611474	[M+H]+	C00195	LMSP02030018	70678862
641.595824	[M+H]+	C00195	LMSP02030066	-
665.632209	[M+H]+	C00195	LMSP02010140	-
669.627124	[M+H]+	C00195	-	-
571.517574	[M+H]+	C00195	LMSP02030015	70678878
597.533224	[M+H]+	C00195	LMSP02010191	-
695.679159	[M+H]+	C00195	LMSP02030005	10417280
862.68391	[M+H]+	C11378	LMPR02010001	5281915
794.62131	[M+H]+	C01967	LMPR02010004	5280473
596.537975	[M+NH4]+	C00641	LMGL02010020	9543688
612.475375	[M+NH4]+	C00641	LMGL02010079	9543745
568.506675	[M+NH4]+	C00641	LMGL02010383	53477951
616.506675	[M+NH4]+	C00641	LMGL02010064	9543730
616.506675	[M+NH4]+	C00641	LMGL02010063	9543729
618.522325	[M+NH4]+	C00641	LMGL02010057	9543723
618.522325	[M+NH4]+	C00641	LMGL02010056	9543722
604.506675	[M+NH4]+	C00641	LMGL02010039	-
590.491025	[M+NH4]+	C00641	LMGL02010032	9543700
648.569275	[M+NH4]+	C00641	LMGL02010090	9543756
620.537975	[M+NH4]+	C00641	LMGL02010049	9543716
620.537975	[M+NH4]+	C00641	LMGL02010050	6441562
606.522325	[M+NH4]+	C00641	LMGL02010007	-
592.506675	[M+NH4]+	C00641	LMGL02010027	9543695
614.491025	[M+NH4]+	C00641	LMGL02010071	9543737
706.647525	[M+NH4]+	C00641	-	53478084
592.506675	[M+NH4]+	C00641	LMGL02010026	9543694
596.537975	[M+NH4]+	C00641	LMGL02010388	53477955
610.553625	[M+NH4]+	C00641	LMGL02010025	9543693
624.569275	[M+NH4]+	C00641	LMGL02010002	102615
624.569275	[M+NH4]+	C00641	LMGL02010395	53477963
652.600575	[M+NH4]+	C00641	LMGL02010008	3246949
538.459725	[M+NH4]+	C00641	LMGL02010379	53477950
568.506675	[M+NH4]+	C00641	LMGL02010001	99931
580.506675	[M+NH4]+	C00641	LMGL02010432	53478004
594.522325	[M+NH4]+	C00641	LMGL02010006	5282283
608.537975	[M+NH4]+	C00641	LMGL02010030	-
622.553625	[M+NH4]+	C00641	LMGL02010043	6443547
650.584925	[M+NH4]+	C00641	LMGL02010081	9543747
580.506675	[M+NH4]+	C00641	LMGL02010014	-
678.616225	[M+NH4]+	C00641	-	-
914.596675	[M+NH4]+	-	-	-
914.596675	[M+NH4]+	-	-	-
942.627975	[M+NH4]+	-	-	-
942.627975	[M+NH4]+	-	-	-
940.612325	[M+NH4]+	-	LMGL05010015	52922093
944.643625	[M+NH4]+	-	-	-
938.596675	[M+NH4]+	-	-	-
936.581025	[M+NH4]+	-	-	-
962.596675	[M+NH4]+	-	-	-
940.612325	[M+NH4]+	-	-	-
916.612325	[M+NH4]+	-	-	-

918.627975	[M+NH4]+	-	-	-
946.659275	[M+NH4]+	-	-	-
890.596675	[M+NH4]+	-	-	-
892.612325	[M+NH4]+	-	-	-
916.612325	[M+NH4]+	-	-	-
759.601304	[M+H]+	-	-	-
759.601304	[M+H]+	-	-	-
761.616954	[M+H]+	-	-	-
763.632604	[M+H]+	-	-	-
735.601304	[M+H]+	-	LMGL00000126	42607374
737.616954	[M+H]+	-	-	-
711.601304	[M+H]+	-	LMGL00000125	42607372
703.538704	[M+H]+	-	-	-
172.14633	[M-H]-	C01571	LMFA01010010	2969
302.22458	[M-H]-	C06428	LMFA01030759	5282846
270.25588	[M-H]-	-	LMFA01010017	10465
284.27153	[M-H]-	C01530	LMFA01010018	5281
340.33413	[M-H]-	C08281	LMFA01010022	8215
368.36543	[M-H]-	C08320	LMFA01010024	11197
368.36543	[M-H]-	-	LMFA01010028	10470
268.24023	[M-H]-	C16536	LMFA01030059	5282747
282.25588	[M-H]-	C00712	LMFA01030061	5282749
310.28718	[M-H]-	C16526	LMFA01030082	5282765
338.31848	[M-H]-	C08316	LMFA01030088	5282771
256.24023	[M-H]-	C00249	LMFA01010001	985
278.22458	[M-H]-	C08364	LMFA01030140	5282815
360.30283	[M-H]-	-	LMFA01030805	-
356.27153	[M-H]-	-	LMFA01030822	5282851
280.24023	[M-H]-	C01595	LMFA01030110	5280450
809.674469	[M+H]+	C01190	LMSP0501AA08	6321360
781.643169	[M+H]+	C01190	-	-
753.611869	[M+H]+	C01190	-	-
725.580569	[M+H]+	C01190	LMSP0501AA27	24779575
697.549269	[M+H]+	C01190	-	-
699.564919	[M+H]+	C01190	LMSP0501AA03	6321355
699.564919	[M+H]+	C01190	LMSP0501AA49	70699230
841.664299	[M+H]+	C01190	-	-
871.711249	[M+H]+	C01190	LMSP05010047	134812542
843.679949	[M+H]+	C01190	LMSP05010058	134812552
829.664299	[M+H]+	C01190	-	-
787.617349	[M+H]+	C01190	LMSP05010044	134812539
731.554749	[M+H]+	C01190	LMSP05010042	134812537
753.611869	[M+H]+	C01190	LMSP0501AA34	52931255
781.643169	[M+H]+	C01190	LMSP0501AA37	52931258
809.674469	[M+H]+	C01190	-	-
697.549269	[M+H]+	C01190	LMSP0501AA33	52931254
815.648649	[M+H]+	C01190	LMSP05010045	134812540
725.580569	[M+H]+	C01190	-	-
497.371639	[M+H]+	-	-	-
495.355989	[M+H]+	-	-	-
471.355989	[M+H]+	-	-	-
501.402939	[M+H]+	-	-	-
487.387289	[M+H]+	-	-	-
473.371639	[M+H]+	-	-	-

499.387289	[M+H]+	-	-	-
432.227692	[M-H]-	C00681	LMGP10050023	52929757
482.243343	[M-H]-	C00681	-	-
434.243343	[M-H]-	C00681	LMGP10050017	50990923
410.24334	[M-H]-	C00681	LMGP10050042	53478599
438.27464	[M-H]-	C00681	LMGP10050043	53478600
436.258993	[M-H]-	C00681	LMGP10050008	5311263
567.332491	[M+H]+	C04230	LMGP01050056	10415542
519.332491	[M+H]+	C04230	LMGP01050034	24779467
491.316841	[M+H]+	C04230	-	-
549.379441	[M+H]+	C04230	LMGP01050047	24779475
535.363791	[M+H]+	C04230	LMGP01050130	52924049
517.316841	[M+H]+	C04230	LMGP01050038	24779469
507.332491	[M+H]+	C04230	LMGP01050002	42607442
493.316841	[M+H]+	C04230	LMGP01050021	24779460
479.301191	[M+H]+	C04230	LMGP01050125	52924041
579.426391	[M+H]+	C04230	LMGP01050053	24779479
467.301191	[M+H]+	C04230	LMGP01050012	460604
551.395091	[M+H]+	C04230	LMGP01050045	24779473
495.332491	[M+H]+	C04230	LMGP01050018	460602
509.348141	[M+H]+	C04230	LMGP01050024	24779463
523.363791	[M+H]+	C04230	LMGP01050026	497299
521.348141	[M+H]+	C04230	LMGP01050029	24779465
475.269891	[M+H]+	-	LMGP02050042	53480927
477.285541	[M+H]+	-	LMGP02050011	52925130
451.269891	[M+H]+	-	LMGP02050037	53480923
465.285541	[M+H]+	-	LMGP02050008	42607464
565.410741	[M+H]+	-	LMGP02050061	53480946
537.379441	[M+H]+	-	LMGP02050054	53480939
509.348141	[M+H]+	-	LMGP02050045	53480930
453.285541	[M+H]+	-	LMGP02050002	9547069
479.301191	[M+H]+	-	LMGP02050004	9547071
510.295772	[M-H]-	-	LMGP04050006	9547135
484.280122	[M-H]-	-	LMGP04050008	42607483
512.311422	[M-H]-	-	LMGP04050009	42607484
508.280122	[M-H]-	-	LMGP04050014	52927437
572.296167	[M-H]-	-	LMGP06050002	42607493
598.311817	[M-H]-	-	LMGP06050005	42607496
596.296167	[M-H]-	-	LMGP06050010	52928605
330.27701	[M+NH4]+	C01885	LMGL01010001	14900
358.30831	[M+NH4]+	C01885	LMGL01010003	24699
356.29266	[M+NH4]+	C01885	LMGL01010004	5283467
354.27701	[M+NH4]+	C01885	LMGL01010006	5283469
354.27701	[M+NH4]+	C01885	-	-
780.57515	[M+NH4]+	-	-	-
752.54385	[M+NH4]+	-	LMGL05010056	-
754.5595	[M+NH4]+	-	-	-
778.5595	[M+NH4]+	-	-	-
774.5282	[M+NH4]+	-	LMGL05010001	5771744
782.5908	[M+NH4]+	-	LMGL05010021	52922099
776.54385	[M+NH4]+	-	LMGL05010024	52922102
754.5595	[M+NH4]+	-	LMGL05010026	52922104
756.57515	[M+NH4]+	-	LMGL05010025	52922103
702.5282	[M+NH4]+	-	LMGL05010049	-

778.5595	[M+NH4]+	-	LMGL05010023	52922101
730.5595	[M+NH4]+	-	-	-
800.54385	[M+NH4]+	-	-	-
796.60645	[M+NH4]+	-	-	-
648.473007	[M-H]-	C00416	LMGP10010012	446066
646.457357	[M-H]-	C00416	LMGP10010911	52929501
672.473007	[M-H]-	C00416	LMGP10010023	9547167
700.504307	[M-H]-	C00416	LMGP10010962	14057128
728.535607	[M-H]-	C00416	LMGP10010198	52928791
670.457357	[M-H]-	C00416	-	-
698.488657	[M-H]-	C00416	LMGP10010216	52928809
696.473007	[M-H]-	C00416	LMGP10010957	46891867
694.457357	[M-H]-	C00416	LMGP10010624	52929217
634.457357	[M-H]-	C00416	LMGP10010912	52929502
704.535607	[M-H]-	C00416	LMGP10010028	447938
674.488657	[M-H]-	C00416	LMGP10010007	9547158
731.546506	[M+H]+	C00157	LMGP01010490	24778618
733.562156	[M+H]+	C00157	LMGP01010739	3082163
759.577806	[M+H]+	C00157	LMGP01010005	5497103
689.499556	[M-H]-	C00350	LMGP02010520	52924223
743.546506	[M-H]-	C00350	LMGP02010039	9546744
691.515206	[M-H]-	C00350	LMGP02010037	445468
663.483906	[M-H]-	C00350	LMGP02010297	446670
717.530856	[M-H]-	C00350	LMGP02010009	5283496
715.515206	[M-H]-	C00350	LMGP02011194	52924895
750.541087	[M-H]-	-	LMGP04010888	52927153
720.494137	[M-H]-	-	LMGP04010882	52927147
748.525437	[M-H]-	-	LMGP04010002	5283509
776.556737	[M-H]-	-	LMGP04010037	24779551
746.509787	[M-H]-	-	LMGP04010877	52927142
774.541087	[M-H]-	-	LMGP04010033	9547127
802.572387	[M-H]-	-	LMGP04010540	52926805
722.509787	[M-H]-	-	LMGP04010986	446440
858.525832	[M-H]-	-	-	-
858.525832	[M-H]-	-	LMGP06010927	52928380
860.541482	[M-H]-	-	LMGP06010931	52928384
832.510182	[M-H]-	-	LMGP06010340	52927793
832.510182	[M-H]-	-	LMGP06010314	52927767
862.557132	[M-H]-	-	-	-
862.557132	[M-H]-	-	LMGP06010956	52928409
848.541482	[M-H]-	-	LMGP06010227	-
864.572782	[M-H]-	-	LMGP06010957	52928410
836.541482	[M-H]-	-	LMGP06010001	5771758
866.588432	[M-H]-	-	LMGP06010942	52928395
838.557132	[M-H]-	-	LMGP06010878	52928331
810.525832	[M-H]-	-	-	-
834.525832	[M-H]-	-	LMGP06010847	52928300
788.535607	[M-H]-	-	-	-
760.504307	[M-H]-	-	-	-
634.457357	[M-H]-	-	-	-
787.536336	[M-H]-	C02737	LMGP03010031	9547091
815.567636	[M-H]-	C02737	LMGP03010956	52926063
803.567636	[M-H]-	C02737	LMGP03010332	52925442
297.266779	[M+H]+	C00836	LMSP01080010	11220228

299.282429	[M+H]+	C00836	LMSP01010001	5280335
271.251129	[M+H]+	C00836	LMSP01040008	14767871
301.298079	[M+H]+	C00836	LMSP01020001	91486
285.230394	[M+H]+	C00836	-	-
315.277344	[M+H]+	C00836	LMSP01030002	14757418
317.292994	[M+H]+	C00836	LMSP01030001	122121
794.5214	[M+NH4]+	-	LMGL05010004	42607399
842.5214	[M+NH4]+	-	-	-
842.5214	[M+NH4]+	-	-	-
766.4901	[M+NH4]+	-	LMGL05010005	42607401
816.50575	[M+NH4]+	-	-	-
846.5527	[M+NH4]+	-	-	-
818.5214	[M+NH4]+	-	-	-
820.53705	[M+NH4]+	-	-	-
806.5214	[M+NH4]+	-	-	-
792.50575	[M+NH4]+	-	LMGL05010006	42607403
844.53705	[M+NH4]+	-	-	-
910.79894	[M+NH4]+	C00422	-	-
924.81459	[M+NH4]+	C00422	-	-
938.83024	[M+NH4]+	C00422	-	131760148
938.83024	[M+NH4]+	C00422	LMGL03011041	9545002
938.83024	[M+NH4]+	C00422	LMGL03011042	25240386
938.83024	[M+NH4]+	C00422	-	-
938.83024	[M+NH4]+	C00422	LMGL03010858	9544819
938.83024	[M+NH4]+	C00422	LMGL03010861	9544822
952.84589	[M+NH4]+	C00422	-	-
966.86154	[M+NH4]+	C00422	LMGL03011425	9545386
966.86154	[M+NH4]+	C00422	LMGL03011423	9545384
966.86154	[M+NH4]+	C00422	-	-
966.86154	[M+NH4]+	C00422	-	-
980.87719	[M+NH4]+	C00422	-	-
994.89284	[M+NH4]+	C00422	-	131761312
910.79894	[M+NH4]+	C00422	LMGL03010545	9544507
824.68939	[M+NH4]+	C00422	LMGL03014382	56938171
824.68939	[M+NH4]+	C00422	LMGL03012781	56936577
838.70504	[M+NH4]+	C00422	LMGL03015166	56938948
838.70504	[M+NH4]+	C00422	LMGL03012961	56936753
952.84589	[M+NH4]+	C00422	-	-
910.79894	[M+NH4]+	C00422	LMGL03010537	9544499
882.76764	[M+NH4]+	C00422	LMGL03010307	9544271
910.79894	[M+NH4]+	C00422	LMGL03010532	9544494
996.90849	[M+NH4]+	C00422	-	-
812.68939	[M+NH4]+	C00422	LMGL03015431	56939213
812.68939	[M+NH4]+	C00422	LMGL03013928	56937718
812.68939	[M+NH4]+	C00422	LMGL03013802	56937592
826.70504	[M+NH4]+	C00422	LMGL03012793	56936587
826.70504	[M+NH4]+	C00422	LMGL03010064	25240359
826.70504	[M+NH4]+	C00422	LMGL03010065	9544033
826.70504	[M+NH4]+	C00422	LMGL03014363	56938152
826.70504	[M+NH4]+	C00422	-	-
840.72069	[M+NH4]+	C00422	LMGL03012907	53481043
910.79894	[M+NH4]+	C00422	LMGL03010544	9544506
840.72069	[M+NH4]+	C00422	LMGL03010091	9544057
854.73634	[M+NH4]+	C00422	LMGL03010145	25240365

868.75199	[M+NH4]+	C00422	LMGL03010190	-
868.75199	[M+NH4]+	C00422	LMGL03010189	9544154
882.76764	[M+NH4]+	C00422	LMGL03010288	25240371
882.76764	[M+NH4]+	C00422	LMGL03010302	9544266
882.76764	[M+NH4]+	C00422	LMGL03010311	9544275
882.76764	[M+NH4]+	C00422	LMGL03010291	9544255
896.78329	[M+NH4]+	C00422	-	-
910.79894	[M+NH4]+	C00422	LMGL03010695	9544657
910.79894	[M+NH4]+	C00422	LMGL03010539	9544501
854.73634	[M+NH4]+	C00422	LMGL03010141	25240363
838.70504	[M+NH4]+	C00422	LMGL03013810	56937600
866.73634	[M+NH4]+	C00422	LMGL03010223	9544188
852.72069	[M+NH4]+	C00422	LMGL03010167	25240367
878.73634	[M+NH4]+	C00422	LMGL03010373	9544336
878.73634	[M+NH4]+	C00422	LMGL03010371	5322095
878.73634	[M+NH4]+	C00422	LMGL03013030	56936821
878.73634	[M+NH4]+	C00422	LMGL03010372	9544335
906.76764	[M+NH4]+	C00422	LMGL03010663	131750594
906.76764	[M+NH4]+	C00422	LMGL03010653	9544615
906.76764	[M+NH4]+	C00422	LMGL03010657	9544619
848.68939	[M+NH4]+	C00422	-	-
848.68939	[M+NH4]+	C00422	LMGL03013481	56937271
876.72069	[M+NH4]+	C00422	LMGL03010420	9544383
876.72069	[M+NH4]+	C00422	LMGL03016218	131760199
904.75199	[M+NH4]+	C00422	LMGL03010722	9544683
874.70504	[M+NH4]+	C00422	LMGL03010471	9544434
874.70504	[M+NH4]+	C00422	LMGL03010502	9544465
902.73634	[M+NH4]+	C00422	LMGL03010815	131760201
872.68939	[M+NH4]+	C00422	LMGL03010527	5462874
928.75199	[M+NH4]+	C00422	LMGL03011453	9545414
870.67374	[M+NH4]+	C00422	LMGL03013042	56936832
898.70504	[M+NH4]+	C00422	LMGL03010954	9544915
898.70504	[M+NH4]+	C00422	LMGL03010947	9544908
968.87719	[M+NH4]+	C00422	LMGL03011339	25240389
864.72069	[M+NH4]+	C00422	LMGL03010253	9544217
850.70504	[M+NH4]+	C00422	LMGL03010192	9544157
850.70504	[M+NH4]+	C00422	LMGL03010214	9544179
850.70504	[M+NH4]+	C00422	LMGL03015732	131755331
852.72069	[M+NH4]+	C00422	LMGL03014245	56938034
852.72069	[M+NH4]+	C00422	-	-
852.72069	[M+NH4]+	C00422	LMGL03015728	56939510
866.73634	[M+NH4]+	C00422	LMGL03010218	-
866.73634	[M+NH4]+	C00422	LMGL03010222	9544187
880.75199	[M+NH4]+	C00422	LMGL03010327	25240373
880.75199	[M+NH4]+	C00422	LMGL03010355	9544319
880.75199	[M+NH4]+	C00422	LMGL03010329	9544293
880.75199	[M+NH4]+	C00422	LMGL03010346	9544310
880.75199	[M+NH4]+	C00422	LMGL03010330	9544294
852.72069	[M+NH4]+	C00422	LMGL03010163	-
908.78329	[M+NH4]+	C00422	LMGL03010602	9544564
908.78329	[M+NH4]+	C00422	LMGL03010596	25240380
908.78329	[M+NH4]+	C00422	LMGL03010591	9544553
908.78329	[M+NH4]+	C00422	LMGL03010595	9544557
936.81459	[M+NH4]+	C00422	LMGL03011124	25240388

936.81459	[M+NH4]+	C00422	LMGL03011116	9545077
936.81459	[M+NH4]+	C00422	LMGL03011123	9545084
936.81459	[M+NH4]+	C00422	LMGL03010929	9544890
964.84589	[M+NH4]+	C00422	-	-
836.68939	[M+NH4]+	C00422	LMGL03012908	56936700
850.70504	[M+NH4]+	C00422	LMGL03010215	9544180
908.78329	[M+NH4]+	C00422	LMGL03010601	9544563
968.87719	[M+NH4]+	C00422	LMGL03011338	9545299
928.84589	[M+NH4]+	C00422	-	-
954.86154	[M+NH4]+	C00422	-	-
888.81459	[M+NH4]+	C00422	LMGL03010188	16058371
888.81459	[M+NH4]+	C00422	LMGL03010205	9544170
874.79894	[M+NH4]+	C00422	LMGL03010119	9544084
860.78329	[M+NH4]+	C00422	LMGL03010096	9544062
860.78329	[M+NH4]+	C00422	LMGL03010095	9544061
860.78329	[M+NH4]+	C00422	LMGL03010085	25240360
846.76764	[M+NH4]+	C00422	LMGL03010051	9544019
888.81459	[M+NH4]+	C00422	-	-
832.75199	[M+NH4]+	C00422	LMGL03010005	5283474
818.73634	[M+NH4]+	C00422	LMGL03015010	56938799
804.72069	[M+NH4]+	C00422	LMGL03014226	56938015
804.72069	[M+NH4]+	C00422	LMGL03010017	9543986
790.70504	[M+NH4]+	C00422	-	-
790.70504	[M+NH4]+	C00422	LMGL03013791	56937581
790.70504	[M+NH4]+	C00422	LMGL03015005	56938794
776.68939	[M+NH4]+	C00422	LMGL03012762	56936558
818.73634	[M+NH4]+	C00422	LMGL03010021	9543990
776.68939	[M+NH4]+	C00422	LMGL03012844	56936638
888.81459	[M+NH4]+	C00422	LMGL03010201	9544166
902.83024	[M+NH4]+	C00422	LMGL03010455	9544418
802.70504	[M+NH4]+	C00422	LMGL03014632	56938421
802.70504	[M+NH4]+	C00422	LMGL03014251	-
802.70504	[M+NH4]+	C00422	LMGL03014227	56938040
802.70504	[M+NH4]+	C00422	LMGL03010018	-
788.68939	[M+NH4]+	C00422	LMGL03012901	56936695
774.67374	[M+NH4]+	C00422	LMGL03014627	56938416
972.90849	[M+NH4]+	C00422	-	-
902.83024	[M+NH4]+	C00422	-	131754165
958.89284	[M+NH4]+	C00422	-	-
944.87719	[M+NH4]+	C00422	-	131755092
930.86154	[M+NH4]+	C00422	-	-
930.86154	[M+NH4]+	C00422	-	131754270
916.84589	[M+NH4]+	C00422	LMGL03010383	9544346
916.84589	[M+NH4]+	C00422	LMGL03014499	-
916.84589	[M+NH4]+	C00422	LMGL03010511	9544474
916.84589	[M+NH4]+	C00422	LMGL03010382	9544345
944.87719	[M+NH4]+	C00422	LMGL03010823	9544784
816.72069	[M+NH4]+	C00422	LMGL03010025	-
776.68939	[M+NH4]+	C00422	LMGL03014221	56938011
762.67374	[M+NH4]+	C00422	LMGL03013681	56937471
848.78329	[M+NH4]+	C00422	LMGL03010041	9544009
834.76764	[M+NH4]+	C00422	LMGL03015058	56938844
834.76764	[M+NH4]+	C00422	LMGL03010004	3246953
820.75199	[M+NH4]+	C00422	LMGL03015009	56938798

820.75199	[M+NH4]+	C00422	LMGL03010019	9543988
806.73634	[M+NH4]+	C00422	LMGL03015006	56938795
806.73634	[M+NH4]+	C00422	LMGL03014225	91865747
862.79894	[M+NH4]+	C00422	LMGL03010069	545690
806.73634	[M+NH4]+	C00422	LMGL03010001	11147
792.72069	[M+NH4]+	C00422	LMGL03012900	56936694
778.70504	[M+NH4]+	C00422	LMGL03012786	91865745
764.68939	[M+NH4]+	C00422	LMGL03014167	56937957
750.67374	[M+NH4]+	C00422	LMGL03013732	56937522
750.67374	[M+NH4]+	C00422	LMGL03012756	91865743
736.65809	[M+NH4]+	C00422	LMGL03013675	56937465
722.64244	[M+NH4]+	C00422	LMGL03013211	56937001
792.72069	[M+NH4]+	C00422	LMGL03014222	56938012
776.68939	[M+NH4]+	C00422	LMGL03013326	56937116
862.79894	[M+NH4]+	C00422	LMGL03010080	9544047
876.81459	[M+NH4]+	C00422	LMGL03010130	9544095
762.67374	[M+NH4]+	C00422	LMGL03014168	56937958
748	[M+NH4]+	C00422	LMGL03012757	56936554
748.65809	[M+NH4]+	C00422	LMGL03013217	56937007
748.65809	[M+NH4]+	C00422	LMGL03013322	56937112
720.62679	[M+NH4]+	C00422	LMGL03013212	56937002
932.87719	[M+NH4]+	C00422	-	-
932.87719	[M+NH4]+	C00422	-	-
876.81459	[M+NH4]+	C00422	LMGL03015132	56938916
918.86154	[M+NH4]+	C00422	LMGL03010335	9544299
918.86154	[M+NH4]+	C00422	LMGL03010459	9544422
904.84589	[M+NH4]+	C00422	-	-
904.84589	[M+NH4]+	C00422	-	131754157
890.83024	[M+NH4]+	C00422	LMGL03010249	9544214
890.83024	[M+NH4]+	C00422	LMGL03014355	56938144
890.83024	[M+NH4]+	C00422	LMGL03010175	9544140
890.83024	[M+NH4]+	C00422	LMGL03010002	11146
918.86154	[M+NH4]+	C00422	-	131754998
968.87719	[M+NH4]+	C00422	-	131758073
816.72069	[M+NH4]+	C00422	LMGL03015011	56938800
816.72069	[M+NH4]+	C00422	LMGL03014298	56938087
884.78329	[M+NH4]+	C00422	LMGL03010250	5497163
870.76764	[M+NH4]+	C00422	LMGL03010164	9544129
856.75199	[M+NH4]+	C00422	LMGL03010125	25240362
856.75199	[M+NH4]+	C00422	LMGL03014370	56938159
856.75199	[M+NH4]+	C00422	LMGL03010118	9544083
856.75199	[M+NH4]+	C00422	LMGL03010121	25240361
842.73634	[M+NH4]+	C00422	LMGL03010077	9544044
884.78329	[M+NH4]+	C00422	LMGL03010252	9544216
842.73634	[M+NH4]+	C00422	LMGL03010075	-
828.72069	[M+NH4]+	C00422	LMGL03014362	56938151
828.72069	[M+NH4]+	C00422	LMGL03010054	25240358
828.72069	[M+NH4]+	C00422	-	-
828.72069	[M+NH4]+	C00422	LMGL03010053	9544021
828.72069	[M+NH4]+	C00422	LMGL03010052	131759416
814.70504	[M+NH4]+	C00422	-	-
800.68939	[M+NH4]+	C00422	LMGL03014228	56938017
842.73634	[M+NH4]+	C00422	LMGL03015146	131750653
800.68939	[M+NH4]+	C00422	LMGL03014252	56938041

884.78329	[M+NH4]+	C00422	LMGL03010271	9544235
884.78329	[M+NH4]+	C00422	LMGL03010267	9544231
940.84589	[M+NH4]+	C00422	LMGL03010785	9544746
940.84589	[M+NH4]+	C00422	LMGL03010788	9544749
940.84589	[M+NH4]+	C00422	LMGL03010976	25240385
940.84589	[M+NH4]+	C00422	LMGL03010969	25240384
926.83024	[M+NH4]+	C00422	-	-
912.81459	[M+NH4]+	C00422	LMGL03010477	9544440
912.81459	[M+NH4]+	C00422	LMGL03010632	9544594
884.78329	[M+NH4]+	C00422	LMGL03010256	25240369
912.81459	[M+NH4]+	C00422	LMGL03010485	9544448
912.81459	[M+NH4]+	C00422	LMGL03010483	131750373
912.81459	[M+NH4]+	C00422	LMGL03010484	9544447
912.81459	[M+NH4]+	C00422	LMGL03010478	25240376
898.79894	[M+NH4]+	C00422	LMGL03013031	-
898.79894	[M+NH4]+	C00422	LMGL03010344	-
884.78329	[M+NH4]+	C00422	LMGL03010262	9544226
884.78329	[M+NH4]+	C00422	-	-
912.81459	[M+NH4]+	C00422	LMGL03010627	9544589
816.72069	[M+NH4]+	C00422	LMGL03012732	56936530
998.92414	[M+NH4]+	C00422	-	-
970.89284	[M+NH4]+	C00422	LMGL03011272	9545233
886.79894	[M+NH4]+	C00422	LMGL03010220	25240368
886.79894	[M+NH4]+	C00422	LMGL03010230	9544195
886.79894	[M+NH4]+	C00422	LMGL03010235	9544200
886.79894	[M+NH4]+	C00422	LMGL03010234	9544199
886.79894	[M+NH4]+	C00422	LMGL03010217	16058372
872.78329	[M+NH4]+	C00422	LMGL03010143	9544108
872.78329	[M+NH4]+	C00422	LMGL03010138	9544103
900.81459	[M+NH4]+	C00422	LMGL03015160	131754360
858.76764	[M+NH4]+	C00422	LMGL03010115	9544080
858.76764	[M+NH4]+	C00422	LMGL03010100	6442384
844.75199	[M+NH4]+	C00422	LMGL03010063	9544031
844.75199	[M+NH4]+	C00422	LMGL03010061	-
830.73634	[M+NH4]+	C00422	LMGL03010044	25240356
830.73634	[M+NH4]+	C00422	LMGL03014259	56938048
830.73634	[M+NH4]+	C00422	LMGL03010043	9544011
816.72069	[M+NH4]+	C00422	LMGL03010024	9543993
858.76764	[M+NH4]+	C00422	LMGL03010104	9544069
998.92414	[M+NH4]+	C00422	-	131758069
900.81459	[M+NH4]+	C00422	LMGL03016144	-
914.83024	[M+NH4]+	C00422	LMGL03010434	25240375
970.89284	[M+NH4]+	C00422	-	131758067
956.87719	[M+NH4]+	C00422	-	-
942.86154	[M+NH4]+	C00422	LMGL03011273	9545234
942.86154	[M+NH4]+	C00422	LMGL03010725	9544686
942.86154	[M+NH4]+	C00422	LMGL03010717	25240382
942.86154	[M+NH4]+	C00422	LMGL03010720	9544681
942.86154	[M+NH4]+	C00422	-	-
900.81459	[M+NH4]+	C00422	-	-
942.86154	[M+NH4]+	C00422	-	131755172
942.86154	[M+NH4]+	C00422	-	131757406
928.84589	[M+NH4]+	C00422	-	131754361
914.83024	[M+NH4]+	C00422	LMGL03010440	9544403

914.83024	[M+NH4]+	C00422	LMGL03010428	25240374
914.83024	[M+NH4]+	C00422	LMGL03010568	9544530
914.83024	[M+NH4]+	C00422	LMGL03010572	9544534
914.83024	[M+NH4]+	C00422	LMGL03010427	9544390
942.86154	[M+NH4]+	C00422	-	131755837
818.73634	[M+NH4]+	C00422	LMGL03010022	9543991

kegg_map
-
-
-
ko00600,ko01100
ko00600,ko01100
ko00600,ko01100
ko00600,ko01100
ko00600,ko01100
ko00600,ko01100
ko00600,ko01100
ko00600,ko01100
ko00600,ko01100
ko00600,ko01100
ko00600,ko01100
ko00600,ko01100
ko00600,ko01100
ko00600,ko01100
ko00600,ko01100
ko00600,ko01100
ko00600,ko01100
ko00600,ko01100
ko00600,ko01100
ko00600,ko01100
ko00600,ko01100
ko00600,ko01100
ko00600,ko01100
ko00600,ko01100
ko00600,ko01100
ko00600,ko01100
ko00600,ko01100
ko00600,ko01100
ko00600,ko01100
ko00600,ko01100
ko00600,ko01100
ko00600,ko01100
ko00600,ko01100
ko00600,ko01100
ko00600,ko01100
ko00600,ko01100
ko00600,ko01100
ko00600,ko01100
ko00600,ko01100
ko00600,ko01100
ko00600,ko01100
ko00600,ko01100
ko00600,ko01100
ko00600,ko01100
ko00600,ko01100
ko00600,ko01100
ko00600,ko01100
ko00600,ko01100
ko00600,ko01100
ko00600,ko01100

ko00600,ko01100
ko00600,ko01100
ko00600,ko01100
ko00600,ko01100
ko00600,ko01100
ko00600,ko01100
ko00600,ko01100
ko00600,ko01100
ko00600,ko01100
ko00600,ko01100
-
-
ko00561,ko00562,ko00564,ko01100,ko01110,ko04070
ko00561,ko00562,ko00564,ko01100,ko01110,ko04070
ko00561,ko00562,ko00564,ko01100,ko01110,ko04070
ko00561,ko00562,ko00564,ko01100,ko01110,ko04070
ko00561,ko00562,ko00564,ko01100,ko01110,ko04070
ko00561,ko00562,ko00564,ko01100,ko01110,ko04070
ko00561,ko00562,ko00564,ko01100,ko01110,ko04070
ko00561,ko00562,ko00564,ko01100,ko01110,ko04070
ko00561,ko00562,ko00564,ko01100,ko01110,ko04070
ko00561,ko00562,ko00564,ko01100,ko01110,ko04070
ko00561,ko00562,ko00564,ko01100,ko01110,ko04070
ko00561,ko00562,ko00564,ko01100,ko01110,ko04070
ko00561,ko00562,ko00564,ko01100,ko01110,ko04070
ko00561,ko00562,ko00564,ko01100,ko01110,ko04070
ko00561,ko00562,ko00564,ko01100,ko01110,ko04070
ko00561,ko00562,ko00564,ko01100,ko01110,ko04070
ko00561,ko00562,ko00564,ko01100,ko01110,ko04070
ko00561,ko00562,ko00564,ko01100,ko01110,ko04070
ko00561,ko00562,ko00564,ko01100,ko01110,ko04070
ko00561,ko00562,ko00564,ko01100,ko01110,ko04070
ko00561,ko00562,ko00564,ko01100,ko01110,ko04070
ko00561,ko00562,ko00564,ko01100,ko01110,ko04070
ko00561,ko00562,ko00564,ko01100,ko01110,ko04070
ko00561,ko00562,ko00564,ko01100,ko01110,ko04070
ko00561,ko00562,ko00564,ko01100,ko01110,ko04070
ko00561,ko00562,ko00564,ko01100,ko01110,ko04070
ko00561,ko00562,ko00564,ko01100,ko01110,ko04070
ko00561,ko00562,ko00564,ko01100,ko01110,ko04070
ko00561,ko00562,ko00564,ko01100,ko01110,ko04070
ko00561,ko00562,ko00564,ko01100,ko01110,ko04070
ko00561,ko00562,ko00564,ko01100,ko01110,ko04070
-
-
-
-
-
-
-
-
-
-
-

-
-
-
-
-
-
-
-
-
-
-
-
-
ko00061,ko01100
ko01040
-
ko00061,ko01040,ko01100
ko00073,ko01040
ko01040
-
-
ko00061,ko00073,ko01040
ko01040
ko01040
ko00061,ko00062,ko00071,ko00073,ko01040,ko01100,ko01212
-
-
-
ko00591,ko01040,ko01100
ko00600,ko01100
ko00600,ko01100
ko00600,ko01100
ko00600,ko01100
ko00600,ko01100
ko00600,ko01100
ko00600,ko01100
ko00600,ko01100
ko00600,ko01100
ko00600,ko01100
ko00600,ko01100
ko00600,ko01100
ko00600,ko01100
ko00600,ko01100
ko00600,ko01100
ko00600,ko01100
ko00600,ko01100
ko00600,ko01100
ko00600,ko01100
-
-
-
-
-
-

-
ko00561,ko00564,ko01100,ko01110
ko00561,ko00564,ko01100,ko01110
ko00561,ko00564,ko01100,ko01110
ko00561,ko00564,ko01100,ko01110
ko00561,ko00564,ko01100,ko01110
ko00561,ko00564,ko01100,ko01110
ko00564
ko00564
ko00564
ko00564
ko00564
ko00564
ko00564
ko00564
ko00564
ko00564
ko00564
ko00564
ko00564
ko00564
ko00564
ko00564
-
-
-
-
-
-
-
-
-
-
-
-
-
-
-
-
ko00561,ko01100
ko00561,ko01100
ko00561,ko01100
ko00561,ko01100
ko00561,ko01100
-
-
-
-
-
-
-
-
-
-

-
-
-
-
ko00561,ko00564,ko01100,ko01110,ko04070
ko00561,ko00564,ko01100,ko01110,ko04070
ko00561,ko00564,ko01100,ko01110,ko04070
ko00561,ko00564,ko01100,ko01110,ko04070
ko00561,ko00564,ko01100,ko01110,ko04070
ko00561,ko00564,ko01100,ko01110,ko04070
ko00561,ko00564,ko01100,ko01110,ko04070
ko00561,ko00564,ko01100,ko01110,ko04070
ko00561,ko00564,ko01100,ko01110,ko04070
ko00561,ko00564,ko01100,ko01110,ko04070
ko00561,ko00564,ko01100,ko01110,ko04070
ko00561,ko00564,ko01100,ko01110,ko04070
ko00564,ko00590,ko00591,ko00592,ko01100,ko01110
ko00564,ko00590,ko00591,ko00592,ko01100,ko01110
ko00564,ko00590,ko00591,ko00592,ko01100,ko01110
ko00563,ko00564,ko01100,ko01110,ko04136
ko00563,ko00564,ko01100,ko01110,ko04136
ko00563,ko00564,ko01100,ko01110,ko04136
ko00563,ko00564,ko01100,ko01110,ko04136
ko00563,ko00564,ko01100,ko01110,ko04136
ko00563,ko00564,ko01100,ko01110,ko04136
-
-
-
-
-
-
-
-
-
-
-
-
-
-
-
-
-
-
-
-
-
-
-
-
-
ko00260,ko00564,ko01100,ko01110
ko00260,ko00564,ko01100,ko01110
ko00260,ko00564,ko01100,ko01110
ko00600,ko01100

ko00600,ko01100
ko00600,ko01100
ko00600,ko01100
ko00600,ko01100
ko00600,ko01100
ko00600,ko01100
-
-
-
-
-
-
-
-
-
-
-
ko00561,ko01100
ko00561,ko01100
ko00561,ko01100
ko00561,ko01100
ko00561,ko01100
ko00561,ko01100
ko00561,ko01100
ko00561,ko01100
ko00561,ko01100
ko00561,ko01100
ko00561,ko01100
ko00561,ko01100
ko00561,ko01100
ko00561,ko01100
ko00561,ko01100
ko00561,ko01100
ko00561,ko01100
ko00561,ko01100
ko00561,ko01100
ko00561,ko01100
ko00561,ko01100
ko00561,ko01100
ko00561,ko01100
ko00561,ko01100
ko00561,ko01100
ko00561,ko01100
ko00561,ko01100
ko00561,ko01100
ko00561,ko01100
ko00561,ko01100
ko00561,ko01100
ko00561,ko01100
ko00561,ko01100
ko00561,ko01100
ko00561,ko01100
ko00561,ko01100
ko00561,ko01100

ko00561,ko01100
ko00561,ko01100
ko00561,ko01100
ko00561,ko01100
ko00561,ko01100
ko00561,ko01100
ko00561,ko01100
ko00561,ko01100
ko00561,ko01100
ko00561,ko01100
ko00561,ko01100
ko00561,ko01100
ko00561,ko01100
ko00561,ko01100
ko00561,ko01100
ko00561,ko01100
ko00561,ko01100
ko00561,ko01100
ko00561,ko01100
ko00561,ko01100
ko00561,ko01100
ko00561,ko01100
ko00561,ko01100
ko00561,ko01100
ko00561,ko01100
ko00561,ko01100
ko00561,ko01100
ko00561,ko01100
ko00561,ko01100
ko00561,ko01100
ko00561,ko01100
ko00561,ko01100
ko00561,ko01100
ko00561,ko01100
ko00561,ko01100
ko00561,ko01100
ko00561,ko01100
ko00561,ko01100
ko00561,ko01100
ko00561,ko01100
ko00561,ko01100
ko00561,ko01100
ko00561,ko01100
ko00561,ko01100
ko00561,ko01100
ko00561,ko01100
ko00561,ko01100
ko00561,ko01100
ko00561,ko01100
ko00561,ko01100
ko00561,ko01100
ko00561,ko01100
ko00561,ko01100
ko00561,ko01100

ko00561,ko01100
ko00561,ko01100
ko00561,ko01100
ko00561,ko01100
ko00561,ko01100
ko00561,ko01100
ko00561,ko01100
ko00561,ko01100
ko00561,ko01100
ko00561,ko01100
ko00561,ko01100
ko00561,ko01100
ko00561,ko01100
ko00561,ko01100
ko00561,ko01100
ko00561,ko01100
ko00561,ko01100
ko00561,ko01100
ko00561,ko01100
ko00561,ko01100
ko00561,ko01100
ko00561,ko01100
ko00561,ko01100
ko00561,ko01100
ko00561,ko01100
ko00561,ko01100
ko00561,ko01100
ko00561,ko01100
ko00561,ko01100
ko00561,ko01100
ko00561,ko01100
ko00561,ko01100
ko00561,ko01100
ko00561,ko01100
ko00561,ko01100
ko00561,ko01100
ko00561,ko01100
ko00561,ko01100
ko00561,ko01100
ko00561,ko01100
ko00561,ko01100
ko00561,ko01100
ko00561,ko01100
ko00561,ko01100
ko00561,ko01100
ko00561,ko01100
ko00561,ko01100
ko00561,ko01100
ko00561,ko01100
ko00561,ko01100
ko00561,ko01100
ko00561,ko01100
ko00561,ko01100
ko00561,ko01100

ko00561,ko01100
ko00561,ko01100
ko00561,ko01100
ko00561,ko01100
ko00561,ko01100
ko00561,ko01100
ko00561,ko01100
ko00561,ko01100
ko00561,ko01100
ko00561,ko01100
ko00561,ko01100
ko00561,ko01100
ko00561,ko01100
ko00561,ko01100
ko00561,ko01100
ko00561,ko01100
ko00561,ko01100
ko00561,ko01100
ko00561,ko01100
ko00561,ko01100
ko00561,ko01100
ko00561,ko01100
ko00561,ko01100
ko00561,ko01100
ko00561,ko01100
ko00561,ko01100
ko00561,ko01100
ko00561,ko01100
ko00561,ko01100
ko00561,ko01100
ko00561,ko01100
ko00561,ko01100
ko00561,ko01100
ko00561,ko01100
ko00561,ko01100
ko00561,ko01100
ko00561,ko01100
ko00561,ko01100
ko00561,ko01100
ko00561,ko01100
ko00561,ko01100
ko00561,ko01100
ko00561,ko01100
ko00561,ko01100
ko00561,ko01100
ko00561,ko01100
ko00561,ko01100
ko00561,ko01100
ko00561,ko01100
ko00561,ko01100
ko00561,ko01100
ko00561,ko01100
ko00561,ko01100
ko00561,ko01100

ko00561,ko01100
ko00561,ko01100
ko00561,ko01100
ko00561,ko01100
ko00561,ko01100
ko00561,ko01100
ko00561,ko01100
ko00561,ko01100
ko00561,ko01100
ko00561,ko01100
ko00561,ko01100
ko00561,ko01100
ko00561,ko01100
ko00561,ko01100
ko00561,ko01100
ko00561,ko01100
ko00561,ko01100
ko00561,ko01100
ko00561,ko01100
ko00561,ko01100
ko00561,ko01100
ko00561,ko01100
ko00561,ko01100
ko00561,ko01100
ko00561,ko01100
ko00561,ko01100
ko00561,ko01100
ko00561,ko01100
ko00561,ko01100
ko00561,ko01100
ko00561,ko01100
ko00561,ko01100
ko00561,ko01100
ko00561,ko01100
ko00561,ko01100
ko00561,ko01100
ko00561,ko01100
ko00561,ko01100
ko00561,ko01100
ko00561,ko01100
ko00561,ko01100
ko00561,ko01100
ko00561,ko01100
ko00561,ko01100
ko00561,ko01100
ko00561,ko01100
ko00561,ko01100
ko00561,ko01100
ko00561,ko01100
ko00561,ko01100
ko00561,ko01100
ko00561,ko01100
ko00561,ko01100
ko00561,ko01100

ko00561,ko01100
ko00561,ko01100
ko00561,ko01100
ko00561,ko01100
ko00561,ko01100
ko00561,ko01100
