# Supplementary material for: Lipidomic Profiling and Storage-Induced Changes in Cassava Flour Using LC-MS/MS
Source: Foods. 2024 Sep 25;13(19):3039. doi: 10.3390/foods13193039 (PMC11475662; doi:10.3390/foods13193039)
Supplement: Supplementary file 1 [file foods-13-03039-s001.zip › Supplementary material.pdf]

Table S1 Lipid molecular species in SC9 and GR10

Index	Compounds	Class I	Class II	Ionization model
Lipid-B-P-0973	ADGGA((O-16:0)18:1_18:2)	GL	ADGGA	[M+NH4]+
Lipid-B-P-0967	ADGGA((O-16:0)16:0_18:2)	GL	ADGGA	[M+NH4]+
Lipid-B-P-0965	ADGGA((O-16:0)16:0_18:1)	GL	ADGGA	[M+NH4]+
Lipid-B-P-0023	Cer(d18:0/24:0)	SP	Cer	[M+H]+
Lipid-B-P-0040	Cer(d18:1/18:1)	SP	Cer	[M+H]+
Lipid-B-P-0039	Cer(d18:1/16:1)	SP	Cer	[M+H]+
Lipid-B-P-0037	Cer(d18:1/26:0)	SP	Cer	[M+H]+
Lipid-B-P-0035	Cer(d18:1/24:0)	SP	Cer	[M+H]+
Lipid-B-P-0034	Cer(d18:1/23:0)	SP	Cer	[M+H]+
Lipid-B-P-0033	Cer(d18:1/22:0)	SP	Cer	[M+H]+
Lipid-B-P-0032	Cer(d18:1/21:0)	SP	Cer	[M+H]+
Lipid-B-P-0022	Cer(d18:0/22:0)	SP	Cer	[M+H]+
Lipid-B-P-0021	Cer(d18:0/20:0)	SP	Cer	[M+H]+
Lipid-B-P-0020	Cer(d18:0/18:0)	SP	Cer	[M+H]+
Lipid-B-P-0019	Cer(d18:0/16:0)	SP	Cer	[M+H]+
Lipid-B-P-0014	Cer(d18:0/24:0(2OH))	SP	Cer	[M+H]+
Lipid-B-P-0041	Cer(d18:1/20:1)	SP	Cer	[M+H]+
Lipid-B-P-0042	Cer(d18:1/22:1)	SP	Cer	[M+H]+
Lipid-B-P-0036	Cer(d18:1/25:0)	SP	Cer	[M+H]+
Lipid-B-P-0044	Cer(d18:1/26:1)	SP	Cer	[M+H]+
Lipid-B-P-0069	Cer(d18:2/26:3)	SP	Cer	[M+H]+
Lipid-B-P-0068	Cer(d18:2/18:3)	SP	Cer	[M+H]+
Lipid-B-P-0043	Cer(d18:1/24:1)	SP	Cer	[M+H]+
Lipid-B-P-0066	Cer(d18:2/24:1)	SP	Cer	[M+H]+
Lipid-B-P-0065	Cer(d18:2/22:1)	SP	Cer	[M+H]+
Lipid-B-P-0064	Cer(d18:2/20:1)	SP	Cer	[M+H]+
Lipid-B-P-0063	Cer(d18:2/18:1)	SP	Cer	[M+H]+
Lipid-B-P-0067	Cer(d18:2/18:2)	SP	Cer	[M+H]+
Lipid-B-P-0061	Cer(d18:2/26:0)	SP	Cer	[M+H]+
Lipid-B-P-0060	Cer(d18:2/24:0)	SP	Cer	[M+H]+
Lipid-B-P-0059	Cer(d18:2/23:0)	SP	Cer	[M+H]+
Lipid-B-P-0058	Cer(d18:2/22:0)	SP	Cer	[M+H]+
Lipid-B-P-0053	Cer(d18:2/16:0)	SP	Cer	[M+H]+
Lipid-B-P-0062	Cer(d18:2/16:1)	SP	Cer	[M+H]+
Lipid-B-P-0089	Cer(t18:1/23:0(2OH))	SP	Cert	[M+H]+
Lipid-B-P-0091	Cer(t18:1/25:0(2OH))	SP	Cert	[M+H]+
Lipid-B-P-0092	Cer(t18:1/26:0(2OH))	SP	Cert	[M+H]+
Lipid-B-P-0093	Cer(t18:1/27:0(2OH))	SP	Cert	[M+H]+
Lipid-B-P-0098	Cer(t17:0/24:0)	SP	Cert	[M+H]+
Lipid-B-P-0104	Cer(t18:0/22:0)	SP	Cert	[M+H]+
Lipid-B-P-0103	Cer(t18:0/18:0)	SP	Cert	[M+H]+
Lipid-B-P-0111	Cer(t18:1/26:0)	SP	Cert	[M+H]+
Lipid-B-P-0073	Cer(t18:0/18:0(2OH))	SP	Cert	[M+H]+

Lipid-B-P-0105	Cer(t18:0/24:0)	SP	Cert	[M+H]+
Lipid-B-P-0088	Cer(t18:1/22:0(2OH))	SP	Cert	[M+H]+
Lipid-B-P-0102	Cer(t18:0/16:0)	SP	Cert	[M+H]+
Lipid-B-P-0087	Cer(t18:1/20:0(2OH))	SP	Cert	[M+H]+
Lipid-B-P-0074	Cer(t18:0/20:0(2OH))	SP	Cert	[M+H]+
Lipid-B-P-0085	Cer(t18:1/16:0(2OH))	SP	Cert	[M+H]+
Lipid-B-P-0084	Cer(t20:0/24:0(2OH))	SP	Cert	[M+H]+
Lipid-B-P-0083	Cer(t18:0/26:1(2OH))	SP	Cert	[M+H]+
Lipid-B-P-0081	Cer(t18:0/24:1(2OH))	SP	Cert	[M+H]+
Lipid-B-P-0080	Cer(t18:0/26:0(2OH))	SP	Cert	[M+H]+
Lipid-B-P-0079	Cer(t18:0/25:0(2OH))	SP	Cert	[M+H]+
Lipid-B-P-0078	Cer(t18:0/24:0(2OH))	SP	Cert	[M+H]+
Lipid-B-P-0077	Cer(t18:0/23:0(2OH))	SP	Cert	[M+H]+
Lipid-B-P-0076	Cer(t18:0/22:0(2OH))	SP	Cert	[M+H]+
Lipid-B-P-0075	Cer(t18:0/21:0(2OH))	SP	Cert	[M+H]+
Lipid-B-P-0110	Cer(t18:1/24:0)	SP	Cert	[M+H]+
Lipid-B-P-0071	Cer(t17:0/24:0(2OH))	SP	Cert	[M+H]+
Lipid-B-P-0072	Cer(t18:0/16:0(2OH))	SP	Cert	[M+H]+
Lipid-B-P-0086	Cer(t18:1/18:0(2OH))	SP	Cert	[M+H]+
Lipid-B-P-0106	Cer(t18:0/26:0)	SP	Cert	[M+H]+
Lipid-B-P-1287	Coenzyme Q10	PR	CoQ	[M+H]+
Lipid-B-P-1286	Coenzyme Q9	PR	CoQ	[M+H]+
Lipid-B-P-0325	DG(16:0_18:0)	GL	DG	[M+NH4]+
Lipid-B-P-0428	DG(18:3_18:3)	GL	DG	[M+NH4]+
Lipid-B-P-0324	DG(14:0_18:0)	GL	DG	[M+NH4]+
Lipid-B-P-0405	DG(18:1_18:3)	GL	DG	[M+NH4]+
Lipid-B-P-0403	DG(18:2_18:2)	GL	DG	[M+NH4]+
Lipid-B-P-0387	DG(18:0_18:3)	GL	DG	[M+NH4]+
Lipid-B-P-0385	DG(18:1_18:2)	GL	DG	[M+NH4]+
Lipid-B-P-0381	DG(17:1_18:2)	GL	DG	[M+NH4]+
Lipid-B-P-0379	DG(16:0_18:3)	GL	DG	[M+NH4]+
Lipid-B-P-0367	DG(18:1_20:1)	GL	DG	[M+NH4]+
Lipid-B-P-0366	DG(18:1_18:1)	GL	DG	[M+NH4]+
Lipid-B-P-0365	DG(18:0_18:2)	GL	DG	[M+NH4]+
Lipid-B-P-0362	DG(17:1_18:1)	GL	DG	[M+NH4]+
Lipid-B-P-0361	DG(16:0_18:2)	GL	DG	[M+NH4]+
Lipid-B-P-0416	DG(18:2_18:3)	GL	DG	[M+NH4]+
Lipid-B-P-0355	DG(18:1_24:0)	GL	DG	[M+NH4]+
Lipid-B-P-0360	DG(16:1_18:1)	GL	DG	[M+NH4]+
Lipid-B-P-0326	DG(14:0_20:0)	GL	DG	[M+NH4]+
Lipid-B-P-0327	DG(17:0_18:0)	GL	DG	[M+NH4]+
Lipid-B-P-0328	DG(18:0_18:0)	GL	DG	[M+NH4]+
Lipid-B-P-0330	DG(14:0_22:0)	GL	DG	[M+NH4]+
Lipid-B-P-0333	DG(20:0_18:0)	GL	DG	[M+NH4]+
Lipid-B-P-0341	DG(14:0_16:1)	GL	DG	[M+NH4]+
Lipid-B-P-0323	DG(16:0_16:0)	GL	DG	[M+NH4]+
Lipid-B-P-0345	DG(15:0_18:1)	GL	DG	[M+NH4]+
Lipid-B-P-0347	DG(16:0_18:1)	GL	DG	[M+NH4]+
Lipid-B-P-0348	DG(17:0_18:1)	GL	DG	[M+NH4]+

Lipid-B-P-0351	DG(18:0_18:1)	GL	DG	[M+NH4]+
Lipid-B-P-0353	DG(18:1_20:0)	GL	DG	[M+NH4]+
Lipid-B-P-0344	DG(16:0_17:1)	GL	DG	[M+NH4]+
Lipid-B-P-0354	DG(18:1_22:0)	GL	DG	[M+NH4]+
Lipid-B-P-1170	DGDG(16:0_18:3)	GL	DGDG	[M+NH4]+
Lipid-B-P-1171	DGDG(16:1_18:2)	GL	DGDG	[M+NH4]+
Lipid-B-P-1174	DGDG(18:0_18:3)	GL	DGDG	[M+NH4]+
Lipid-B-P-1175	DGDG(18:1_18:2)	GL	DGDG	[M+NH4]+
Lipid-B-P-1181	DGDG(18:2_18:2)	GL	DGDG	[M+NH4]+
Lipid-B-P-1166	DGDG(18:0_18:2)	GL	DGDG	[M+NH4]+
Lipid-B-P-1190	DGDG(18:2_18:3)	GL	DGDG	[M+NH4]+
Lipid-B-P-1196	DGDG(18:3_18:3)	GL	DGDG	[M+NH4]+
Lipid-B-P-1203	DGDG(18:2_20:5)	GL	DGDG	[M+NH4]+
Lipid-B-P-1180	DGDG(18:1_18:3)	GL	DGDG	[M+NH4]+
Lipid-B-P-1165	DGDG(16:1_18:1)	GL	DGDG	[M+NH4]+
Lipid-B-P-1157	DGDG(16:0_18:1)	GL	DGDG	[M+NH4]+
Lipid-B-P-1158	DGDG(16:0_20:1)	GL	DGDG	[M+NH4]+
Lipid-B-P-1156	DGDG(14:0_18:1)	GL	DGDG	[M+NH4]+
Lipid-B-P-1154	DGDG(16:0_16:0)	GL	DGDG	[M+NH4]+
Lipid-B-P-1164	DGDG(16:0_18:2)	GL	DGDG	[M+NH4]+
Lipid-B-P-1044	DGTS(18:2_18:2)	GL	DGTS	[M+H]+
Lipid-B-P-1043	DGTS(18:1_18:3)	GL	DGTS	[M+H]+
Lipid-B-P-1036	DGTS(18:1_18:2)	GL	DGTS	[M+H]+
Lipid-B-P-1027	DGTS(18:1_18:1)	GL	DGTS	[M+H]+
Lipid-B-P-1025	DGTS(16:0_18:2)	GL	DGTS	[M+H]+
Lipid-B-P-1018	DGTS(16:0_18:1)	GL	DGTS	[M+H]+
Lipid-B-P-1012	DGTS(16:0_16:0)	GL	DGTS	[M+H]+
Lipid-B-P-1040	DGTS(14:0_18:4)	GL	DGTS	[M+H]+
Lipid-B-N-0001	FFA(10:0)	FA	FFA	[M-H]-
Lipid-B-N-0047	FFA(20:5)	FA	FFA	[M-H]-
Lipid-B-N-0008	FFA(17:0)	FA	FFA	[M-H]-
Lipid-B-N-0009	FFA(18:0)	FA	FFA	[M-H]-
Lipid-B-N-0012	FFA(22:0)	FA	FFA	[M-H]-
Lipid-B-N-0013	FFA(24:0)	FA	FFA	[M-H]-
Lipid-B-N-0015	FFA(28:0)	FA	FFA	[M-H]-
Lipid-B-N-0027	FFA(17:1)	FA	FFA	[M-H]-
Lipid-B-N-0028	FFA(18:1)	FA	FFA	[M-H]-
Lipid-B-N-0030	FFA(20:1)	FA	FFA	[M-H]-
Lipid-B-N-0031	FFA(22:1)	FA	FFA	[M-H]-
Lipid-B-N-0007	FFA(16:0)	FA	FFA	[M-H]-
Lipid-B-N-0039	FFA(18:3)	FA	FFA	[M-H]-
Lipid-B-N-0046	FFA(24:4)	FA	FFA	[M-H]-
Lipid-B-N-0051	FFA(24:6)	FA	FFA	[M-H]-
Lipid-B-N-0034	FFA(18:2)	FA	FFA	[M-H]-
Lipid-B-P-0160	HexCer(d18:1/24:1)	SP	HexCer	[M+H]+
Lipid-B-P-0159	HexCer(d18:1/22:1)	SP	HexCer	[M+H]+
Lipid-B-P-0158	HexCer(d18:1/20:1)	SP	HexCer	[M+H]+
Lipid-B-P-0157	HexCer(d18:1/18:1)	SP	HexCer	[M+H]+
Lipid-B-P-0156	HexCer(d18:1/16:1)	SP	HexCer	[M+H]+

Lipid-B-P-0146	HexCer(d18:1/16:0)	SP	HexCer	[M+H]+
Lipid-B-P-0139	HexCer(d16:1/18:0)	SP	HexCer	[M+H]+
Lipid-B-P-0121	HexCer(t18:1/24:1(2OH))	SP	HexCer	[M+H]+
Lipid-B-P-0117	HexCer(t18:1/26:0(2OH))	SP	HexCer	[M+H]+
Lipid-B-P-0116	HexCer(t18:1/24:0(2OH))	SP	HexCer	[M+H]+
Lipid-B-P-0115	HexCer(t18:1/23:0(2OH))	SP	HexCer	[M+H]+
Lipid-B-P-0113	HexCer(t18:1/20:0(2OH))	SP	HexCer	[M+H]+
Lipid-B-P-0112	HexCer(t18:1/16:0(2OH))	SP	HexCer	[M+H]+
Lipid-B-P-0164	HexCer(d18:2/20:0)	SP	HexCer	[M+H]+
Lipid-B-P-0165	HexCer(d18:2/22:0)	SP	HexCer	[M+H]+
Lipid-B-P-0166	HexCer(d18:2/24:0)	SP	HexCer	[M+H]+
Lipid-B-P-0162	HexCer(d18:2/16:0)	SP	HexCer	[M+H]+
Lipid-B-P-0114	HexCer(t18:1/22:0(2OH))	SP	HexCer	[M+H]+
Lipid-B-P-0163	HexCer(d18:2/18:0)	SP	HexCer	[M+H]+
Lipid-B-P-1105	LDGTS(18:2)	GL	LDGTS	[M+H]+
Lipid-B-P-1107	LDGTS(18:3)	GL	LDGTS	[M+H]+
Lipid-B-P-1100	LDGTS(16:1)	GL	LDGTS	[M+H]+
Lipid-B-P-1096	LDGTS(18:0)	GL	LDGTS	[M+H]+
Lipid-B-P-1095	LDGTS(17:0)	GL	LDGTS	[M+H]+
Lipid-B-P-1094	LDGTS(16:0)	GL	LDGTS	[M+H]+
Lipid-B-P-1102	LDGTS(18:1)	GL	LDGTS	[M+H]+
Lipid-B-N-0455	LPA(18:3)	GP	LPA	[M-H]-
Lipid-B-N-0459	LPA(22:5)	GP	LPA	[M-H]-
Lipid-B-N-0453	LPA(18:2)	GP	LPA	[M-H]-
Lipid-B-N-0448	LPA(16:0)	GP	LPA	[M-H]-
Lipid-B-N-0449	LPA(18:0)	GP	LPA	[M-H]-
Lipid-B-N-0451	LPA(18:1)	GP	LPA	[M-H]-
Lipid-B-P-0200	LPC(22:6)	GP	LPC	[M+H]+
Lipid-B-P-0189	LPC(18:2)	GP	LPC	[M+H]+
Lipid-B-P-0188	LPC(16:2)	GP	LPC	[M+H]+
Lipid-B-P-0185	LPC(20:1)	GP	LPC	[M+H]+
Lipid-B-P-0184	LPC(19:1)	GP	LPC	[M+H]+
Lipid-B-P-0193	LPC(18:3)	GP	LPC	[M+H]+
Lipid-B-P-0182	LPC(17:1)	GP	LPC	[M+H]+
Lipid-B-P-0181	LPC(16:1)	GP	LPC	[M+H]+
Lipid-B-P-0180	LPC(15:1)	GP	LPC	[M+H]+
Lipid-B-P-0176	LPC(22:0)	GP	LPC	[M+H]+
Lipid-B-P-0169	LPC(14:0)	GP	LPC	[M+H]+
Lipid-B-P-0175	LPC(20:0)	GP	LPC	[M+H]+
Lipid-B-P-0171	LPC(16:0)	GP	LPC	[M+H]+
Lipid-B-P-0172	LPC(17:0)	GP	LPC	[M+H]+
Lipid-B-P-0173	LPC(18:0)	GP	LPC	[M+H]+
Lipid-B-P-0183	LPC(18:1)	GP	LPC	[M+H]+
Lipid-B-P-0218	LPE(18:3)	GP	LPE	[M+H]+
Lipid-B-P-0214	LPE(18:2)	GP	LPE	[M+H]+
Lipid-B-P-0207	LPE(16:1)	GP	LPE	[M+H]+
Lipid-B-P-0208	LPE(17:1)	GP	LPE	[M+H]+
Lipid-B-P-0206	LPE(24:0)	GP	LPE	[M+H]+
Lipid-B-P-0205	LPE(22:0)	GP	LPE	[M+H]+

Lipid-B-P-0204	LPE(20:0)	GP	LPE	[M+H]+
Lipid-B-P-0201	LPE(16:0)	GP	LPE	[M+H]+
Lipid-B-P-0209	LPE(18:1)	GP	LPE	[M+H]+
Lipid-B-N-0056	LPG(18:1)	GP	LPG	[M-H]-
Lipid-B-N-0052	LPG(16:0)	GP	LPG	[M-H]-
Lipid-B-N-0053	LPG(18:0)	GP	LPG	[M-H]-
Lipid-B-N-0058	LPG(18:2)	GP	LPG	[M-H]-
Lipid-B-N-0066	LPI(16:0)	GP	LPI	[M-H]-
Lipid-B-N-0069	LPI(18:1)	GP	LPI	[M-H]-
Lipid-B-N-0071	LPI(18:2)	GP	LPI	[M-H]-
Lipid-B-P-0457	MG(16:0)	GL	MG	[M+NH4]+
Lipid-B-P-0458	MG(18:0)	GL	MG	[M+NH4]+
Lipid-B-P-0463	MG(18:1)	GL	MG	[M+NH4]+
Lipid-B-P-0465	MG(18:2)	GL	MG	[M+NH4]+
Lipid-B-P-0468	MG(18:3)	GL	MG	[M+NH4]+
Lipid-B-P-1235	MGDG(18:1_18:2)	GL	MGDG	[M+NH4]+
Lipid-B-P-1232	MGDG(16:0_18:3)	GL	MGDG	[M+NH4]+
Lipid-B-P-1224	MGDG(16:1_18:1)	GL	MGDG	[M+NH4]+
Lipid-B-P-1246	MGDG(18:1_18:3)	GL	MGDG	[M+NH4]+
Lipid-B-P-1269	MGDG(18:3_18:3)	GL	MGDG	[M+NH4]+
Lipid-B-P-1226	MGDG(18:1_18:1)	GL	MGDG	[M+NH4]+
Lipid-B-P-1260	MGDG(18:2_18:3)	GL	MGDG	[M+NH4]+
Lipid-B-P-1223	MGDG(16:0_18:2)	GL	MGDG	[M+NH4]+
Lipid-B-P-1217	MGDG(16:0_18:1)	GL	MGDG	[M+NH4]+
Lipid-B-P-1212	MGDG(14:0_16:0)	GL	MGDG	[M+NH4]+
Lipid-B-P-1247	MGDG(18:2_18:2)	GL	MGDG	[M+NH4]+
Lipid-B-P-1213	MGDG(16:0_16:0)	GL	MGDG	[M+NH4]+
Lipid-B-P-1275	MGDG(18:2_20:5)	GL	MGDG	[M+NH4]+
Lipid-B-P-1227	MGDG(18:1_19:1)	GL	MGDG	[M+NH4]+
Lipid-B-N-0463	PA(16:0_16:0)	GP	PA	[M-H]-
Lipid-B-N-0467	PA(16:0_16:1)	GP	PA	[M-H]-
Lipid-B-N-0474	PA(16:0_18:2)	GP	PA	[M-H]-
Lipid-B-N-0475	PA(18:0_18:2)	GP	PA	[M-H]-
Lipid-B-N-0476	PA(18:2_20:0)	GP	PA	[M-H]-
Lipid-B-N-0479	PA(18:3_16:0)	GP	PA	[M-H]-
Lipid-B-N-0480	PA(18:2_18:1)	GP	PA	[M-H]-
Lipid-B-N-0485	PA(18:1_18:3)	GP	PA	[M-H]-
Lipid-B-N-0489	PA(18:2_18:3)	GP	PA	[M-H]-
Lipid-B-N-0462	PA(16:0_15:0)	GP	PA	[M-H]-
Lipid-B-N-0465	PA(18:0_18:0)	GP	PA	[M-H]-
Lipid-B-N-0468	PA(16:0_18:1)	GP	PA	[M-H]-
Lipid-B-N-0113	PC(14:0_18:1)	GP	PC	[M+H]+
Lipid-B-N-0105	PC(18:0_14:0)	GP	PC	[M+H]+
Lipid-B-N-0115	PC(16:0_18:1)	GP	PC	[M+H]+
Lipid-B-N-0201	PE(16:1_16:0)	GP	PE	[M-H]-
Lipid-B-N-0220	PE(18:1_18:1)	GP	PE	[M-H]-
Lipid-B-N-0195	PE(16:0_16:0)	GP	PE	[M-H]-
Lipid-B-N-0194	PE(16:0_14:0)	GP	PE	[M-H]-
Lipid-B-N-0203	PE(16:0_18:1)	GP	PE	[M-H]-

Lipid-B-N-0218	PE(18:2_16:0)	GP	PE	[M-H]-
Lipid-B-N-0291	PG(18:0_16:0)	GP	PG	[M-H]-
Lipid-B-N-0294	PG(18:1_14:0)	GP	PG	[M-H]-
Lipid-B-N-0296	PG(16:0_18:1)	GP	PG	[M-H]-
Lipid-B-N-0299	PG(18:0_18:1)	GP	PG	[M-H]-
Lipid-B-N-0304	PG(18:2_16:0)	GP	PG	[M-H]-
Lipid-B-N-0306	PG(18:1_18:1)	GP	PG	[M-H]-
Lipid-B-N-0309	PG(20:1_18:1)	GP	PG	[M-H]-
Lipid-B-N-0289	PG(16:0_16:0)	GP	PG	[M-H]-
Lipid-B-N-0384	PI(18:1_18:3)	GP	PI	[M-H]-
Lipid-B-N-0382	PI(18:2_18:2)	GP	PI	[M-H]-
Lipid-B-N-0374	PI(18:1_18:2)	GP	PI	[M-H]-
Lipid-B-N-0372	PI(18:3_16:0)	GP	PI	[M-H]-
Lipid-B-N-0371	PI(18:2_16:1)	GP	PI	[M-H]-
Lipid-B-N-0364	PI(18:1_18:1)	GP	PI	[M-H]-
Lipid-B-N-0363	PI(18:0_18:2)	GP	PI	[M-H]-
Lipid-B-N-0361	PI(17:1_18:1)	GP	PI	[M-H]-
Lipid-B-N-0351	PI(18:0_18:1)	GP	PI	[M-H]-
Lipid-B-N-0348	PI(16:0_18:1)	GP	PI	[M-H]-
Lipid-B-N-0342	PI(16:0_20:0)	GP	PI	[M-H]-
Lipid-B-N-0341	PI(16:0_18:0)	GP	PI	[M-H]-
Lipid-B-N-0340	PI(16:0_16:0)	GP	PI	[M-H]-
Lipid-B-N-0359	PI(18:2_16:0)	GP	PI	[M-H]-
Lipid-B-N-0523	PMeOH(20:2_22:5)	GP	PMeOH	[M-H]-
Lipid-B-N-0522	PMeOH(20:2_20:5)	GP	PMeOH	[M-H]-
Lipid-B-N-0506	PMeOH(14:0_16:0)	GP	PMeOH	[M-H]-
Lipid-B-N-0427	PS(18:0_18:2)	GP	PS	[M-H]-
Lipid-B-N-0429	PS(18:1_20:1)	GP	PS	[M-H]-
Lipid-B-N-0420	PS(18:1_19:0)	GP	PS	[M-H]-
Lipid-B-P-0008	SPH(d18:2)	SP	SPH	[M+H]+
Lipid-B-P-0007	SPH(d18:1)	SP	SPH	[M+H]+
Lipid-B-P-0006	SPH(d16:1)	SP	SPH	[M+H]+
Lipid-B-P-0005	SPH(d18:0)	SP	SPH	[M+H]+
Lipid-B-P-0003	PhytoSph(d16:2)	SP	SPH	[M+H]+
Lipid-B-P-0002	PhytoSph(d18:1)	SP	SPH	[M+H]+
Lipid-B-P-0001	PhytoSph(d18:0)	SP	SPH	[M+H]+
Lipid-B-P-1116	SQDG(16:0_16:0)	GL	SQDG	[M+NH4]+
Lipid-B-P-1141	SQDG(18:2_18:2)	GL	SQDG	[M+NH4]+
Lipid-B-P-1140	SQDG(18:1_18:3)	GL	SQDG	[M+NH4]+
Lipid-B-P-1115	SQDG(14:0_16:0)	GL	SQDG	[M+NH4]+
Lipid-B-P-1135	SQDG(16:0_18:3)	GL	SQDG	[M+NH4]+
Lipid-B-P-1129	SQDG(18:1_18:1)	GL	SQDG	[M+NH4]+
Lipid-B-P-1128	SQDG(16:0_18:2)	GL	SQDG	[M+NH4]+
Lipid-B-P-1122	SQDG(16:0_18:1)	GL	SQDG	[M+NH4]+
Lipid-B-P-1121	SQDG(16:0_17:1)	GL	SQDG	[M+NH4]+
Lipid-B-P-1120	SQDG(16:0_16:1)	GL	SQDG	[M+NH4]+
Lipid-B-P-1136	SQDG(18:1_18:2)	GL	SQDG	[M+NH4]+
Lipid-B-P-0734	TG(20:0_18:2_18:2)	GL	TG	[M+NH4]+
Lipid-B-P-0735	TG(18:2_21:0_18:2)	GL	TG	[M+NH4]+

Lipid-B-P-0736	TG(18:1_22:1_18:2)	GL	TG	[M+NH4]+
Lipid-B-P-0738	TG(18:1_18:3_22:0)	GL	TG	[M+NH4]+
Lipid-B-P-0739	TG(18:2_18:2_22:0)	GL	TG	[M+NH4]+
Lipid-B-P-0740	TG(20:1_20:1_18:2)	GL	TG	[M+NH4]+
Lipid-B-P-0741	TG(18:3_20:0_20:1)	GL	TG	[M+NH4]+
Lipid-B-P-0744	TG(18:1_20:1_20:2)	GL	TG	[M+NH4]+
Lipid-B-P-0745	TG(17:1_18:1_24:2)	GL	TG	[M+NH4]+
Lipid-B-P-0751	TG(18:3_20:0_22:1)	GL	TG	[M+NH4]+
Lipid-B-P-0749	TG(18:1_20:2_22:1)	GL	TG	[M+NH4]+
Lipid-B-P-0753	TG(18:1_24:0_18:3)	GL	TG	[M+NH4]+
Lipid-B-P-0754	TG(18:2_24:0_18:2)	GL	TG	[M+NH4]+
Lipid-B-P-0755	TG(25:0_18:2_18:2)	GL	TG	[M+NH4]+
Lipid-B-P-0756	TG(20:1_24:1_18:2)	GL	TG	[M+NH4]+
Lipid-B-P-0733	TG(18:1_18:1_20:2)	GL	TG	[M+NH4]+
Lipid-B-P-0766	TG(14:0_18:2_18:3)	GL	TG	[M+NH4]+
Lipid-B-P-0770	TG(14:0_14:0_22:5)	GL	TG	[M+NH4]+
Lipid-B-P-0775	TG(15:0_18:2_18:3)	GL	TG	[M+NH4]+
Lipid-B-P-0776	TG(15:1_18:2_18:2)	GL	TG	[M+NH4]+
Lipid-B-P-0746	TG(23:0_18:2_18:2)	GL	TG	[M+NH4]+
Lipid-B-P-0731	TG(18:0_18:3_20:1)	GL	TG	[M+NH4]+
Lipid-B-P-0723	TG(16:0_18:2_20:2)	GL	TG	[M+NH4]+
Lipid-B-P-0729	TG(18:1_18:3_20:0)	GL	TG	[M+NH4]+
Lipid-B-P-0687	TG(18:1_18:2_26:0)	GL	TG	[M+NH4]+
Lipid-B-P-0701	TG(15:1_17:1_17:2)	GL	TG	[M+NH4]+
Lipid-B-P-0702	TG(13:0_18:1_18:3)	GL	TG	[M+NH4]+
Lipid-B-P-0703	TG(13:0_16:0_20:4)	GL	TG	[M+NH4]+
Lipid-B-P-0704	TG(14:0_18:2_18:2)	GL	TG	[M+NH4]+
Lipid-B-P-0705	TG(16:1_16:1_18:2)	GL	TG	[M+NH4]+
Lipid-B-P-0706	TG(16:0_16:1_18:3)	GL	TG	[M+NH4]+
Lipid-B-P-0708	TG(14:0_18:1_18:3)	GL	TG	[M+NH4]+
Lipid-B-P-0712	TG(16:0_16:2_18:2)	GL	TG	[M+NH4]+
Lipid-B-P-0713	TG(15:0_18:2_18:2)	GL	TG	[M+NH4]+
Lipid-B-P-0730	TG(18:0_18:2_20:2)	GL	TG	[M+NH4]+
Lipid-B-P-0714	TG(16:0_17:1_18:3)	GL	TG	[M+NH4]+
Lipid-B-P-0717	TG(16:0_18:1_18:3)	GL	TG	[M+NH4]+
Lipid-B-P-0718	TG(17:1_18:1_18:2)	GL	TG	[M+NH4]+
Lipid-B-P-0720	TG(17:0_18:2_18:2)	GL	TG	[M+NH4]+
Lipid-B-P-0721	TG(18:1_18:1_18:2)	GL	TG	[M+NH4]+
Lipid-B-P-0722	TG(16:0_18:3_20:1)	GL	TG	[M+NH4]+
Lipid-B-P-0724	TG(16:0_18:1_20:3)	GL	TG	[M+NH4]+
Lipid-B-P-0725	TG(18:0_18:1_18:3)	GL	TG	[M+NH4]+
Lipid-B-P-0726	TG(18:1_19:1_18:2)	GL	TG	[M+NH4]+
Lipid-B-P-0727	TG(16:0_18:3_22:1)	GL	TG	[M+NH4]+
Lipid-B-P-0728	TG(16:0_20:2_20:2)	GL	TG	[M+NH4]+
Lipid-B-P-0716	TG(16:0_18:2_18:2)	GL	TG	[M+NH4]+
Lipid-B-P-0777	TG(13:0_16:0_22:5)	GL	TG	[M+NH4]+
Lipid-B-P-0787	TG(17:1_18:1_18:3)	GL	TG	[M+NH4]+
Lipid-B-P-0780	TG(16:0_18:2_18:3)	GL	TG	[M+NH4]+
Lipid-B-P-0833	TG(18:1_18:2_18:3)	GL	TG	[M+NH4]+

Lipid-B-P-0835	TG(18:2_18:2_18:2)	GL	TG	[M+NH4]+
Lipid-B-P-0837	TG(18:1_18:1_18:4)	GL	TG	[M+NH4]+
Lipid-B-P-0838	TG(18:0_18:3_18:3)	GL	TG	[M+NH4]+
Lipid-B-P-0841	TG(18:2_18:2_20:2)	GL	TG	[M+NH4]+
Lipid-B-P-0842	TG(18:3_18:3_20:0)	GL	TG	[M+NH4]+
Lipid-B-P-0844	TG(18:2_18:3_20:1)	GL	TG	[M+NH4]+
Lipid-B-P-0861	TG(16:0_18:2_18:5)	GL	TG	[M+NH4]+
Lipid-B-P-0863	TG(12:0_18:1_22:6)	GL	TG	[M+NH4]+
Lipid-B-P-0869	TG(18:2_18:2_18:3)	GL	TG	[M+NH4]+
Lipid-B-P-0870	TG(18:1_18:2_18:4)	GL	TG	[M+NH4]+
Lipid-B-P-0875	TG(18:3_18:3_20:1)	GL	TG	[M+NH4]+
Lipid-B-P-0897	TG(18:2_18:3_18:3)	GL	TG	[M+NH4]+
Lipid-B-P-0899	TG(16:0_18:3_20:5)	GL	TG	[M+NH4]+
Lipid-B-P-0901	TG(18:1_18:2_20:5)	GL	TG	[M+NH4]+
Lipid-B-P-0914	TG(18:3_18:3_18:3)	GL	TG	[M+NH4]+
Lipid-B-P-0918	TG(16:0_20:4_22:5)	GL	TG	[M+NH4]+
Lipid-B-P-0929	TG(18:2_18:4_18:4)	GL	TG	[M+NH4]+
Lipid-B-P-0931	TG(16:3_18:2_22:5)	GL	TG	[M+NH4]+
Lipid-B-P-0934	TG(16:0_20:5_20:5)	GL	TG	[M+NH4]+
Lipid-B-P-0685	TG(18:2_20:1_22:0)	GL	TG	[M+NH4]+
Lipid-B-P-0832	TG(17:0_18:3_18:3)	GL	TG	[M+NH4]+
Lipid-B-P-0829	TG(16:0_18:3_18:3)	GL	TG	[M+NH4]+
Lipid-B-P-0828	TG(16:1_16:1_20:4)	GL	TG	[M+NH4]+
Lipid-B-P-0825	TG(16:0_18:2_18:4)	GL	TG	[M+NH4]+
Lipid-B-P-0781	TG(14:0_16:0_22:5)	GL	TG	[M+NH4]+
Lipid-B-P-0782	TG(16:3_18:1_18:1)	GL	TG	[M+NH4]+
Lipid-B-P-0784	TG(16:0_18:1_18:4)	GL	TG	[M+NH4]+
Lipid-B-P-0786	TG(17:1_18:2_18:2)	GL	TG	[M+NH4]+
Lipid-B-P-0789	TG(17:0_18:2_18:3)	GL	TG	[M+NH4]+
Lipid-B-P-0790	TG(18:1_18:2_18:2)	GL	TG	[M+NH4]+
Lipid-B-P-0791	TG(16:0_18:1_20:4)	GL	TG	[M+NH4]+
Lipid-B-P-0792	TG(18:0_18:2_18:3)	GL	TG	[M+NH4]+
Lipid-B-P-0793	TG(16:0_18:3_20:2)	GL	TG	[M+NH4]+
Lipid-B-P-0795	TG(18:1_18:1_18:3)	GL	TG	[M+NH4]+
Lipid-B-P-0779	TG(16:1_18:2_18:2)	GL	TG	[M+NH4]+
Lipid-B-P-0798	TG(18:1_18:2_20:2)	GL	TG	[M+NH4]+
Lipid-B-P-0800	TG(18:2_18:2_20:1)	GL	TG	[M+NH4]+
Lipid-B-P-0801	TG(18:2_18:3_20:0)	GL	TG	[M+NH4]+
Lipid-B-P-0802	TG(18:1_18:3_20:1)	GL	TG	[M+NH4]+
Lipid-B-P-0804	TG(18:2_18:2_22:1)	GL	TG	[M+NH4]+
Lipid-B-P-0806	TG(18:2_18:3_22:0)	GL	TG	[M+NH4]+
Lipid-B-P-0807	TG(18:1_18:3_22:1)	GL	TG	[M+NH4]+
Lipid-B-P-0809	TG(18:3_20:1_20:1)	GL	TG	[M+NH4]+
Lipid-B-P-0813	TG(24:0_18:2_18:3)	GL	TG	[M+NH4]+
Lipid-B-P-0823	TG(15:0_18:3_18:3)	GL	TG	[M+NH4]+
Lipid-B-P-0824	TG(16:0_16:1_20:5)	GL	TG	[M+NH4]+
Lipid-B-P-0799	TG(18:0_18:3_20:2)	GL	TG	[M+NH4]+
Lipid-B-P-0683	TG(18:1_22:0_20:2)	GL	TG	[M+NH4]+
Lipid-B-P-0609	TG(16:0_23:0_18:2)	GL	TG	[M+NH4]+

Lipid-B-P-0681	TG(23:0_18:1_18:2)	GL	TG	[M+NH4]+
Lipid-B-P-0541	TG(18:0_18:0_18:1)	GL	TG	[M+NH4]+
Lipid-B-P-0540	TG(16:0_18:0_20:1)	GL	TG	[M+NH4]+
Lipid-B-P-0539	TG(17:0_18:0_18:1)	GL	TG	[M+NH4]+
Lipid-B-P-0538	TG(16:0_16:0_20:1)	GL	TG	[M+NH4]+
Lipid-B-P-0537	TG(16:0_16:1_20:0)	GL	TG	[M+NH4]+
Lipid-B-P-0536	TG(16:0_18:0_18:1)	GL	TG	[M+NH4]+
Lipid-B-P-0535	TG(16:0_17:0_18:1)	GL	TG	[M+NH4]+
Lipid-B-P-0542	TG(16:0_20:0_18:1)	GL	TG	[M+NH4]+
Lipid-B-P-0534	TG(16:0_16:0_18:1)	GL	TG	[M+NH4]+
Lipid-B-P-0531	TG(15:0_16:0_18:1)	GL	TG	[M+NH4]+
Lipid-B-P-0530	TG(14:0_16:0_18:1)	GL	TG	[M+NH4]+
Lipid-B-P-0529	TG(16:0_16:0_16:1)	GL	TG	[M+NH4]+
Lipid-B-P-0528	TG(16:0_16:0_15:1)	GL	TG	[M+NH4]+
Lipid-B-P-0527	TG(13:0_16:0_18:1)	GL	TG	[M+NH4]+
Lipid-B-P-0526	TG(15:0_16:0_16:1)	GL	TG	[M+NH4]+
Lipid-B-P-0525	TG(14:0_14:0_18:1)	GL	TG	[M+NH4]+
Lipid-B-P-0533	TG(16:0_16:1_17:0)	GL	TG	[M+NH4]+
Lipid-B-P-0524	TG(14:1_16:0_16:0)	GL	TG	[M+NH4]+
Lipid-B-P-0544	TG(16:0_18:1_20:0)	GL	TG	[M+NH4]+
Lipid-B-P-0546	TG(16:0_18:1_21:0)	GL	TG	[M+NH4]+
Lipid-B-P-0573	TG(14:1_16:0_18:1)	GL	TG	[M+NH4]+
Lipid-B-P-0572	TG(14:0_16:1_18:1)	GL	TG	[M+NH4]+
Lipid-B-P-0571	TG(14:0_16:0_18:2)	GL	TG	[M+NH4]+
Lipid-B-P-0570	TG(16:0_16:1_16:1)	GL	TG	[M+NH4]+
Lipid-B-P-0569	TG(15:0_16:1_16:1)	GL	TG	[M+NH4]+
Lipid-B-P-0566	TG(14:1_16:0_16:1)	GL	TG	[M+NH4]+
Lipid-B-P-0558	TG(16:0_26:0_18:1)	GL	TG	[M+NH4]+
Lipid-B-P-0545	TG(15:0_16:0_24:1)	GL	TG	[M+NH4]+
Lipid-B-P-0557	TG(16:0_25:0_18:1)	GL	TG	[M+NH4]+
Lipid-B-P-0555	TG(16:0_24:0_18:1)	GL	TG	[M+NH4]+
Lipid-B-P-0554	TG(16:0_23:0_18:1)	GL	TG	[M+NH4]+
Lipid-B-P-0553	TG(15:0_24:0_18:1)	GL	TG	[M+NH4]+
Lipid-B-P-0551	TG(16:0_20:0_20:1)	GL	TG	[M+NH4]+
Lipid-B-P-0549	TG(14:0_20:1_22:0)	GL	TG	[M+NH4]+
Lipid-B-P-0548	TG(16:0_18:1_22:0)	GL	TG	[M+NH4]+
Lipid-B-P-0547	TG(18:0_18:1_20:0)	GL	TG	[M+NH4]+
Lipid-B-P-0556	TG(16:0_20:1_22:0)	GL	TG	[M+NH4]+
Lipid-B-P-0575	TG(16:0_16:1_17:1)	GL	TG	[M+NH4]+
Lipid-B-P-0523	TG(14:0_16:0_16:1)	GL	TG	[M+NH4]+
Lipid-B-P-0521	TG(13:0_14:0_18:1)	GL	TG	[M+NH4]+
Lipid-B-P-0494	TG(16:0_17:0_18:0)	GL	TG	[M+NH4]+
Lipid-B-P-0493	TG(15:0_17:0_18:0)	GL	TG	[M+NH4]+
Lipid-B-P-0492	TG(16:0_16:0_18:0)	GL	TG	[M+NH4]+
Lipid-B-P-0491	TG(15:0_16:0_18:0)	GL	TG	[M+NH4]+
Lipid-B-P-0490	TG(16:0_16:0_17:0)	GL	TG	[M+NH4]+
Lipid-B-P-0489	TG(15:0_16:0_17:0)	GL	TG	[M+NH4]+
Lipid-B-P-0488	TG(14:0_16:0_18:0)	GL	TG	[M+NH4]+
Lipid-B-P-0495	TG(16:0_18:0_18:0)	GL	TG	[M+NH4]+

Lipid-B-P-0487	TG(16:0_16:0_16:0)	GL	TG	[M+NH4]+
Lipid-B-P-0485	TG(15:0_16:0_16:0)	GL	TG	[M+NH4]+
Lipid-B-P-0484	TG(14:0_16:0_16:0)	GL	TG	[M+NH4]+
Lipid-B-P-0483	TG(14:0_15:0_16:0)	GL	TG	[M+NH4]+
Lipid-B-P-0482	TG(13:0_15:0_16:0)	GL	TG	[M+NH4]+
Lipid-B-P-0480	TG(14:0_14:0_16:0)	GL	TG	[M+NH4]+
Lipid-B-P-0479	TG(13:0_14:0_16:0)	GL	TG	[M+NH4]+
Lipid-B-P-0478	TG(12:0_14:0_16:0)	GL	TG	[M+NH4]+
Lipid-B-P-0486	TG(14:0_16:0_17:0)	GL	TG	[M+NH4]+
Lipid-B-P-0522	TG(12:0_16:0_18:1)	GL	TG	[M+NH4]+
Lipid-B-P-0496	TG(16:0_16:0_20:0)	GL	TG	[M+NH4]+
Lipid-B-P-0499	TG(17:0_17:0_19:0)	GL	TG	[M+NH4]+
Lipid-B-P-0520	TG(14:0_15:0_16:1)	GL	TG	[M+NH4]+
Lipid-B-P-0519	TG(14:0_14:0_16:1)	GL	TG	[M+NH4]+
Lipid-B-P-0518	TG(12:0_14:0_18:1)	GL	TG	[M+NH4]+
Lipid-B-P-0517	TG(12:0_16:0_16:1)	GL	TG	[M+NH4]+
Lipid-B-P-0515	TG(12:0_14:0_16:1)	GL	TG	[M+NH4]+
Lipid-B-P-0510	TG(16:0_16:0_25:0)	GL	TG	[M+NH4]+
Lipid-B-P-0509	TG(16:0_18:0_23:0)	GL	TG	[M+NH4]+
Lipid-B-P-0497	TG(15:0_18:0_20:0)	GL	TG	[M+NH4]+
Lipid-B-P-0508	TG(16:0_20:0_20:0)	GL	TG	[M+NH4]+
Lipid-B-P-0506	TG(16:0_18:0_22:0)	GL	TG	[M+NH4]+
Lipid-B-P-0505	TG(16:0_16:0_23:0)	GL	TG	[M+NH4]+
Lipid-B-P-0504	TG(15:0_16:0_24:0)	GL	TG	[M+NH4]+
Lipid-B-P-0503	TG(16:0_16:0_22:0)	GL	TG	[M+NH4]+
Lipid-B-P-0502	TG(14:0_18:0_22:0)	GL	TG	[M+NH4]+
Lipid-B-P-0501	TG(16:0_18:0_20:0)	GL	TG	[M+NH4]+
Lipid-B-P-0500	TG(18:0_18:0_18:0)	GL	TG	[M+NH4]+
Lipid-B-P-0507	TG(16:0_16:0_24:0)	GL	TG	[M+NH4]+
Lipid-B-P-0682	TG(24:0_18:1_18:2)	GL	TG	[M+NH4]+
Lipid-B-P-0576	TG(15:0_16:0_18:2)	GL	TG	[M+NH4]+
Lipid-B-P-0578	TG(14:0_17:1_18:1)	GL	TG	[M+NH4]+
Lipid-B-P-0657	TG(18:1_18:1_18:1)	GL	TG	[M+NH4]+
Lipid-B-P-0655	TG(17:0_18:1_18:2)	GL	TG	[M+NH4]+
Lipid-B-P-0654	TG(16:0_18:0_18:3)	GL	TG	[M+NH4]+
Lipid-B-P-0653	TG(14:0_18:1_20:2)	GL	TG	[M+NH4]+
Lipid-B-P-0652	TG(16:1_18:1_18:1)	GL	TG	[M+NH4]+
Lipid-B-P-0651	TG(16:0_18:1_18:2)	GL	TG	[M+NH4]+
Lipid-B-P-0650	TG(16:0_17:0_18:3)	GL	TG	[M+NH4]+
Lipid-B-P-0658	TG(18:0_18:1_18:2)	GL	TG	[M+NH4]+
Lipid-B-P-0647	TG(16:0_17:1_18:2)	GL	TG	[M+NH4]+
Lipid-B-P-0645	TG(14:0_18:1_18:2)	GL	TG	[M+NH4]+
Lipid-B-P-0644	TG(16:0_16:0_18:3)	GL	TG	[M+NH4]+
Lipid-B-P-0643	TG(16:0_16:2_18:1)	GL	TG	[M+NH4]+
Lipid-B-P-0642	TG(16:0_16:1_18:2)	GL	TG	[M+NH4]+
Lipid-B-P-0641	TG(16:1_16:1_18:1)	GL	TG	[M+NH4]+
Lipid-B-P-0640	TG(16:0_15:1_18:2)	GL	TG	[M+NH4]+
Lipid-B-P-0636	TG(14:0_16:0_18:3)	GL	TG	[M+NH4]+
Lipid-B-P-0646	TG(15:0_18:1_18:2)	GL	TG	[M+NH4]+

Lipid-B-P-0635	TG(14:0_16:1_18:2)	GL	TG	[M+NH4]+
Lipid-B-P-0659	TG(16:0_18:1_20:2)	GL	TG	[M+NH4]+
Lipid-B-P-0661	TG(16:0_18:2_20:1)	GL	TG	[M+NH4]+
Lipid-B-P-0678	TG(18:3_20:0_20:0)	GL	TG	[M+NH4]+
Lipid-B-P-0677	TG(18:2_20:0_20:1)	GL	TG	[M+NH4]+
Lipid-B-P-0675	TG(18:1_18:1_22:1)	GL	TG	[M+NH4]+
Lipid-B-P-0674	TG(18:1_18:2_22:0)	GL	TG	[M+NH4]+
Lipid-B-P-0673	TG(21:0_18:1_18:2)	GL	TG	[M+NH4]+
Lipid-B-P-0672	TG(18:0_18:3_20:0)	GL	TG	[M+NH4]+
Lipid-B-P-0671	TG(16:0_18:2_22:1)	GL	TG	[M+NH4]+
Lipid-B-P-0660	TG(18:0_18:0_18:3)	GL	TG	[M+NH4]+
Lipid-B-P-0670	TG(16:0_20:1_20:2)	GL	TG	[M+NH4]+
Lipid-B-P-0668	TG(20:1_18:2_18:0)	GL	TG	[M+NH4]+
Lipid-B-P-0667	TG(18:1_18:1_20:1)	GL	TG	[M+NH4]+
Lipid-B-P-0666	TG(18:1_18:2_20:0)	GL	TG	[M+NH4]+
Lipid-B-P-0665	TG(18:1_18:1_19:1)	GL	TG	[M+NH4]+
Lipid-B-P-0664	TG(17:1_18:1_20:1)	GL	TG	[M+NH4]+
Lipid-B-P-0663	TG(16:0_18:3_20:0)	GL	TG	[M+NH4]+
Lipid-B-P-0662	TG(20:0_18:1_18:2)	GL	TG	[M+NH4]+
Lipid-B-P-0669	TG(16:0_18:3_22:0)	GL	TG	[M+NH4]+
Lipid-B-P-0577	TG(13:0_18:1_18:1)	GL	TG	[M+NH4]+
Lipid-B-P-0622	TG(26:0_18:1_18:1)	GL	TG	[M+NH4]+
Lipid-B-P-0620	TG(18:0_20:1_22:1)	GL	TG	[M+NH4]+
Lipid-B-P-0597	TG(18:0_18:0_18:2)	GL	TG	[M+NH4]+
Lipid-B-P-0595	TG(16:0_18:2_20:0)	GL	TG	[M+NH4]+
Lipid-B-P-0593	TG(16:1_18:0_20:1)	GL	TG	[M+NH4]+
Lipid-B-P-0592	TG(16:0_18:1_20:1)	GL	TG	[M+NH4]+
Lipid-B-P-0591	TG(18:0_18:1_18:1)	GL	TG	[M+NH4]+
Lipid-B-P-0590	TG(17:0_18:0_18:2)	GL	TG	[M+NH4]+
Lipid-B-P-0589	TG(17:0_18:1_18:1)	GL	TG	[M+NH4]+
Lipid-B-P-0598	TG(15:0_18:1_22:1)	GL	TG	[M+NH4]+
Lipid-B-P-0588	TG(16:0_16:0_20:2)	GL	TG	[M+NH4]+
Lipid-B-P-0586	TG(16:0_18:1_18:1)	GL	TG	[M+NH4]+
Lipid-B-P-0585	TG(16:0_17:0_18:2)	GL	TG	[M+NH4]+
Lipid-B-P-0584	TG(16:0_17:1_18:1)	GL	TG	[M+NH4]+
Lipid-B-P-0583	TG(16:0_16:0_18:2)	GL	TG	[M+NH4]+
Lipid-B-P-0582	TG(14:0_16:1_20:1)	GL	TG	[M+NH4]+
Lipid-B-P-0581	TG(16:0_16:1_18:1)	GL	TG	[M+NH4]+
Lipid-B-P-0580	TG(16:1_16:1_17:0)	GL	TG	[M+NH4]+
Lipid-B-P-0587	TG(16:0_18:0_18:2)	GL	TG	[M+NH4]+
Lipid-B-P-0621	TG(24:0_18:1_20:1)	GL	TG	[M+NH4]+
Lipid-B-P-0599	TG(18:0_18:1_19:1)	GL	TG	[M+NH4]+
Lipid-B-P-0601	TG(18:0_18:1_20:1)	GL	TG	[M+NH4]+
Lipid-B-P-0619	TG(24:0_18:1_18:1)	GL	TG	[M+NH4]+
Lipid-B-P-0618	TG(23:0_18:1_18:1)	GL	TG	[M+NH4]+
Lipid-B-P-0617	TG(18:1_20:0_22:1)	GL	TG	[M+NH4]+
Lipid-B-P-0616	TG(18:0_20:0_20:2)	GL	TG	[M+NH4]+
Lipid-B-P-0615	TG(18:2_20:0_20:0)	GL	TG	[M+NH4]+
Lipid-B-P-0614	TG(18:1_20:0_20:1)	GL	TG	[M+NH4]+

Lipid-B-P-0613	TG(16:0_24:0_18:2)	GL	TG	[M+NH4]+
Lipid-B-P-0600	TG(16:0_21:0_18:2)	GL	TG	[M+NH4]+
Lipid-B-P-0612	TG(16:0_18:1_24:1)	GL	TG	[M+NH4]+
Lipid-B-P-0610	TG(22:0_18:1_18:1)	GL	TG	[M+NH4]+
Lipid-B-P-0608	TG(15:0_18:1_24:1)	GL	TG	[M+NH4]+
Lipid-B-P-0607	TG(18:0_18:0_20:2)	GL	TG	[M+NH4]+
Lipid-B-P-0606	TG(18:1_18:1_20:0)	GL	TG	[M+NH4]+
Lipid-B-P-0604	TG(16:0_18:2_22:0)	GL	TG	[M+NH4]+
Lipid-B-P-0603	TG(16:0_18:1_22:1)	GL	TG	[M+NH4]+
Lipid-B-P-0602	TG(18:0_18:2_20:0)	GL	TG	[M+NH4]+
Lipid-B-P-0611	TG(18:0_22:0_18:2)	GL	TG	[M+NH4]+
Lipid-B-P-0532	TG(16:0_16:0_17:1)	GL	TG	[M+NH4]+

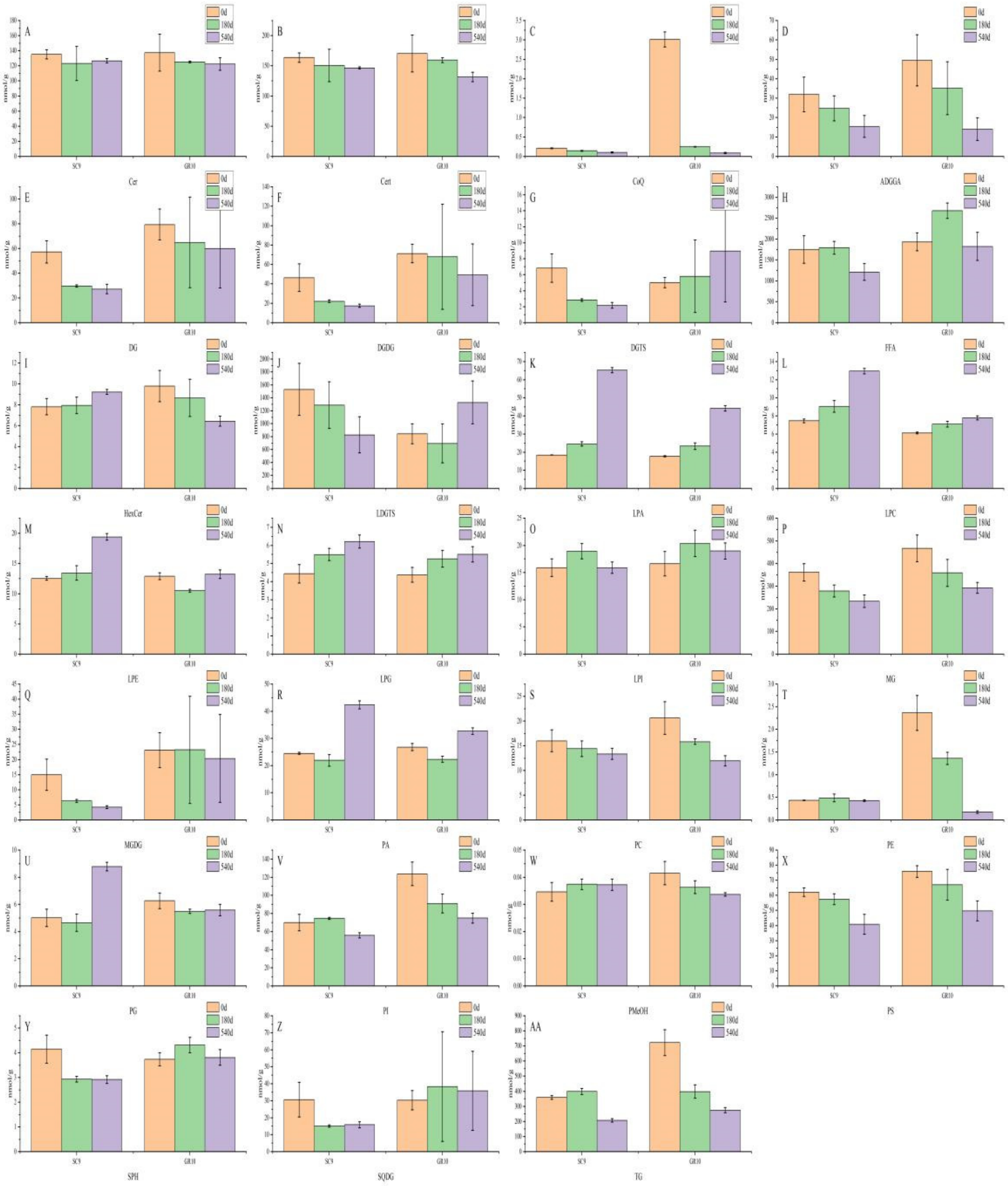

Fig. S1. The content of Cer (A), Cert (B), CoQ (C), ADGGA (D), DG (E), DGDG (F), DGTS (G), FFA (H), HesCer (I), LDGTS (J), LPA (K), LPC (L), LPE (M), LPG (N), LPI (O), MG (P), MGDG (Q), PA (R), PC (S), PE (T), PG (U), PI (V), PMeOH (W), PS (X), SPH (Y), SQDG (Z), TG (AA) in SC9 and GR10 during storage (n = 3).
