# Supplementary figures and images for: Lipidomic Profiling and Storage-Induced Changes in Cassava Flour Using LC-MS/MS
Source: Foods. 2024 Sep 25;13(19):3039. doi: 10.3390/foods13193039 (PMC11475662; doi:10.3390/foods13193039)

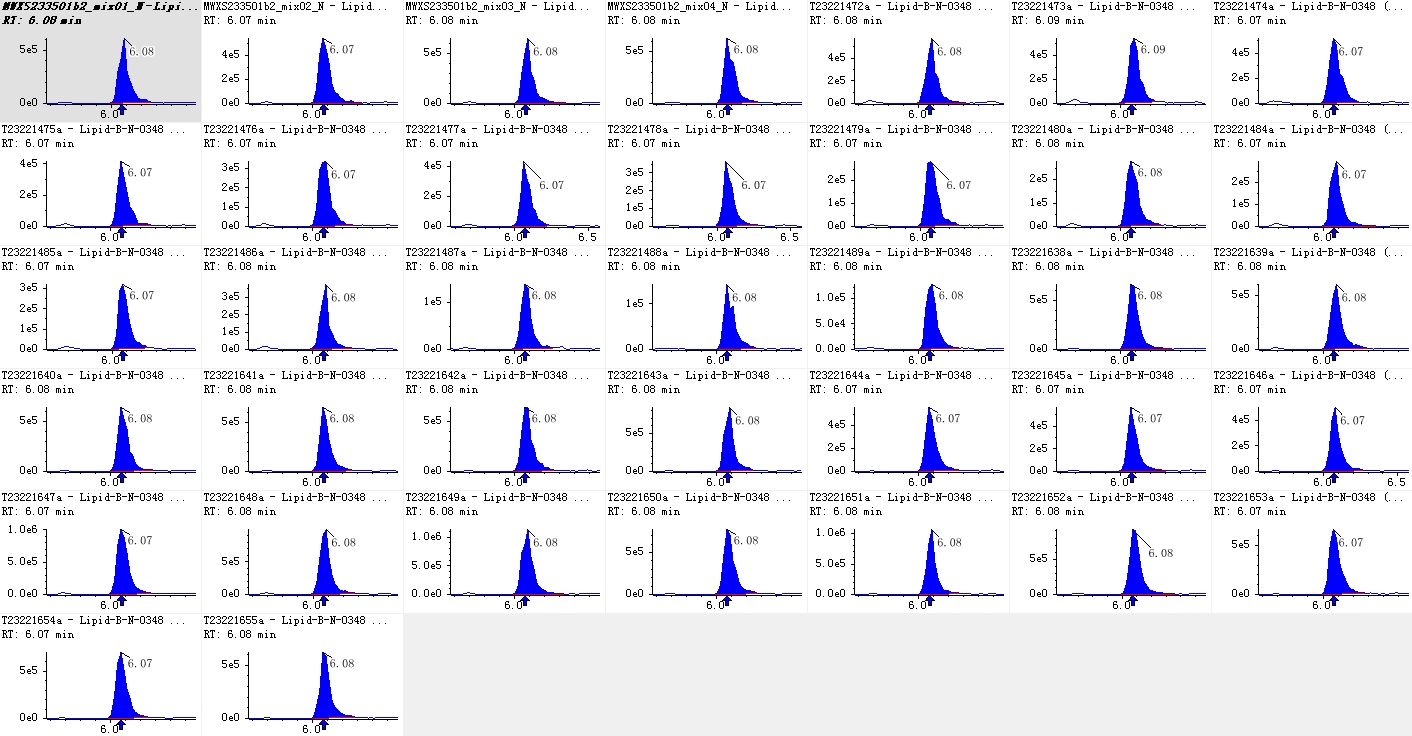

Supplement: Supplementary file 1 [file foods-13-03039-s001.zip › 0.Data/QC/MWXS-23-3501-b2_Integral_correction_diagram-N.png]

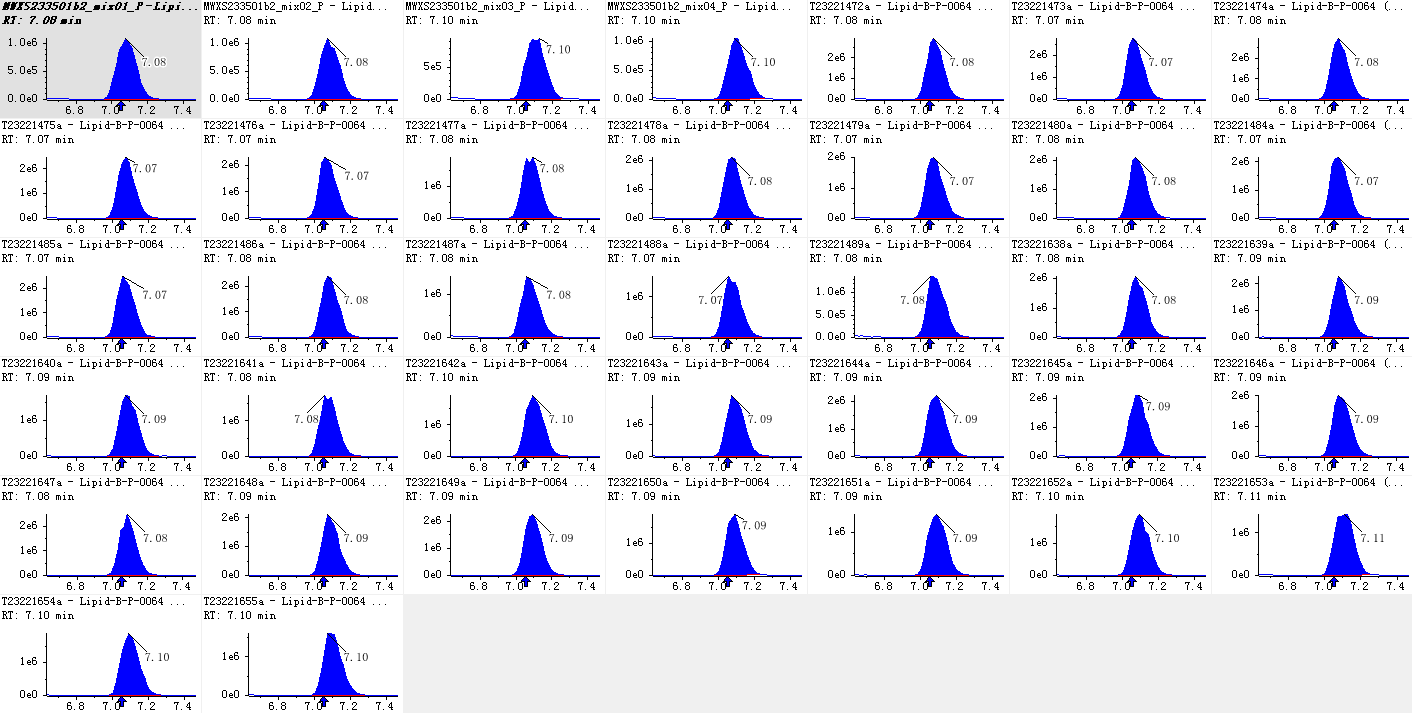

Supplement: Supplementary file 1 [file foods-13-03039-s001.zip › 0.Data/QC/MWXS-23-3501-b2_Integral_correction_diagram-P.png]

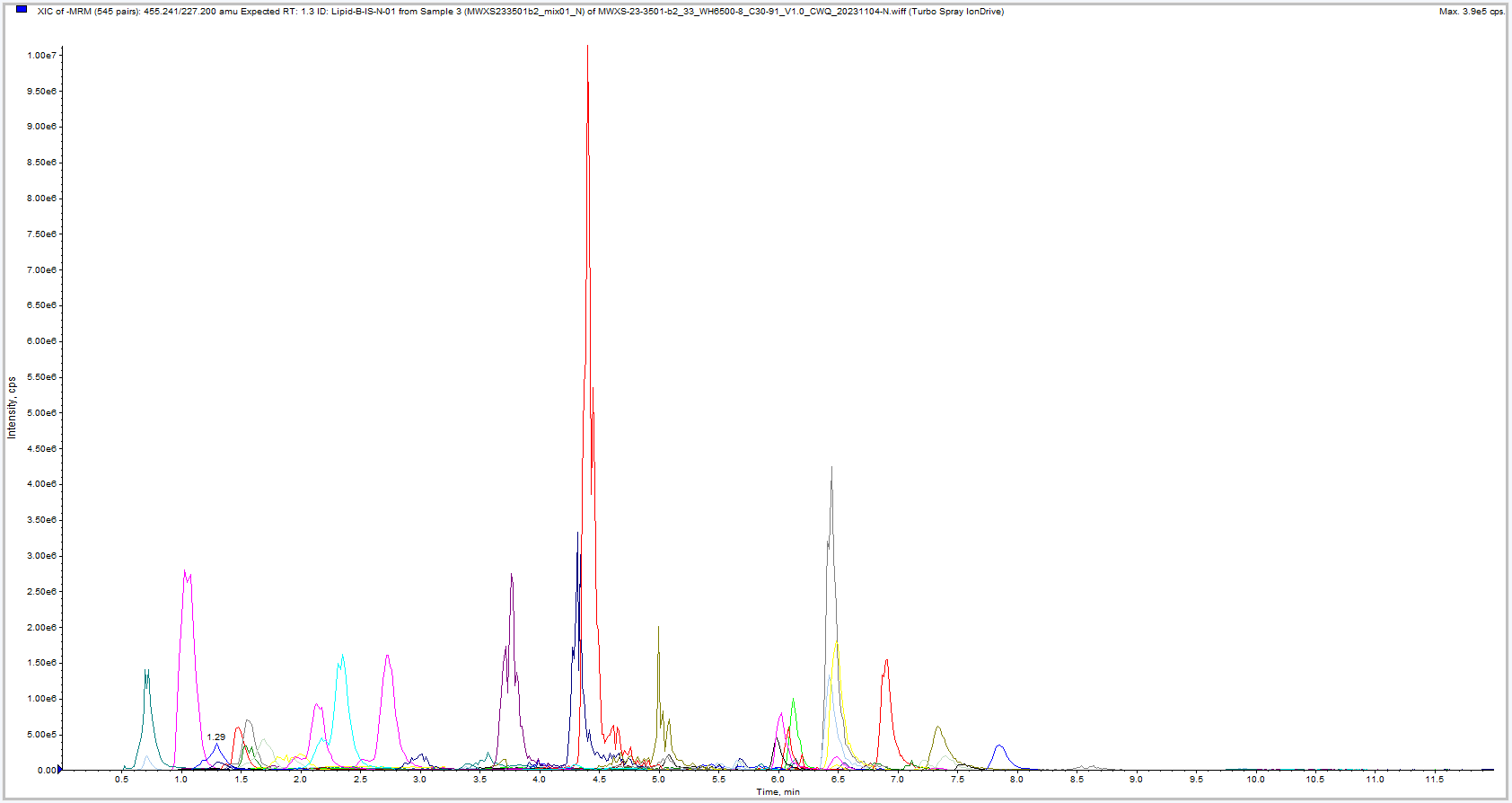

Supplement: Supplementary file 1 [file foods-13-03039-s001.zip › 0.Data/QC/MWXS-23-3501-b2_MRM_detection_of_multimodal_maps-N.png]

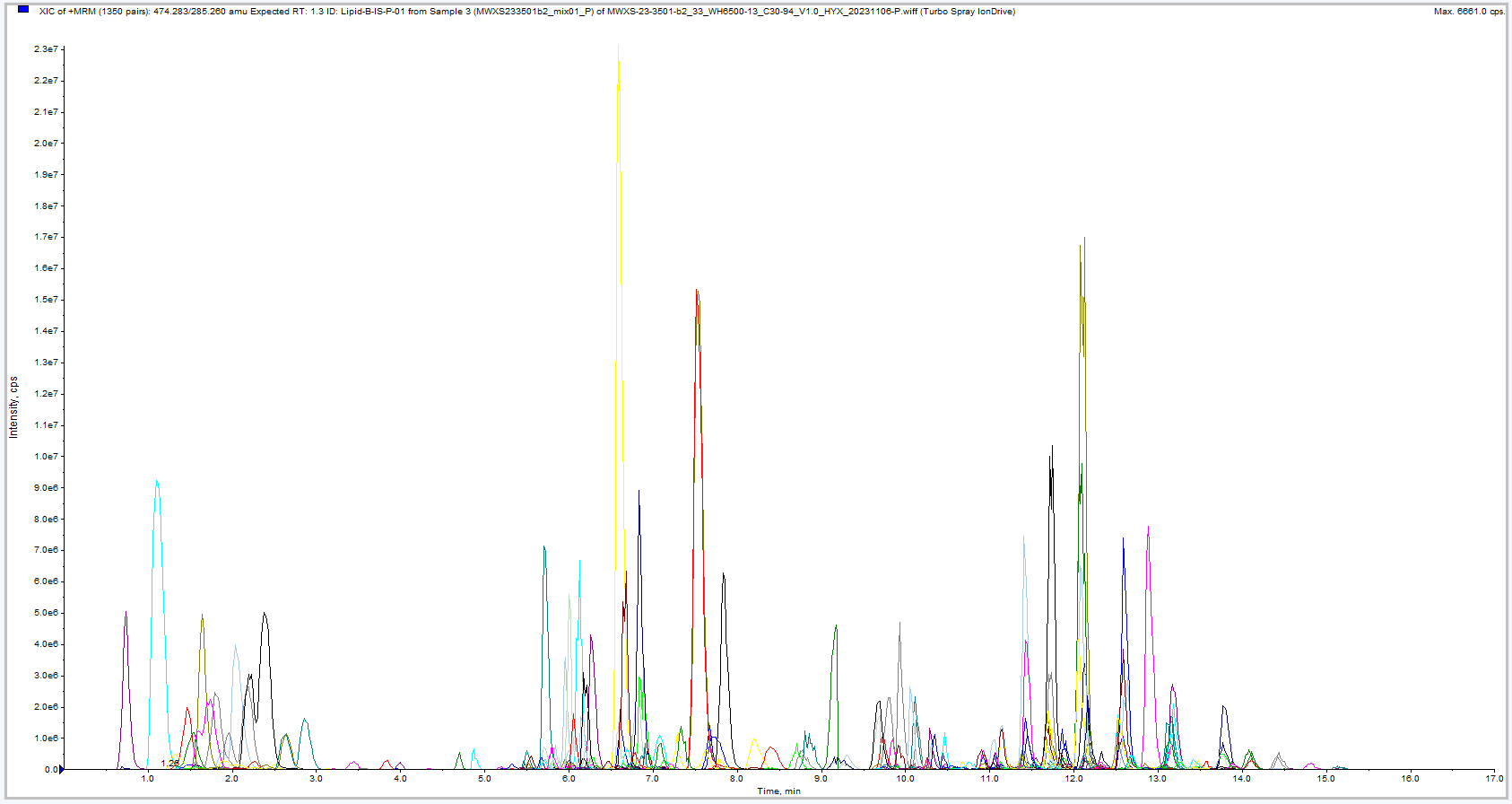

Supplement: Supplementary file 1 [file foods-13-03039-s001.zip › 0.Data/QC/MWXS-23-3501-b2_MRM_detection_of_multimodal_maps-P.png]

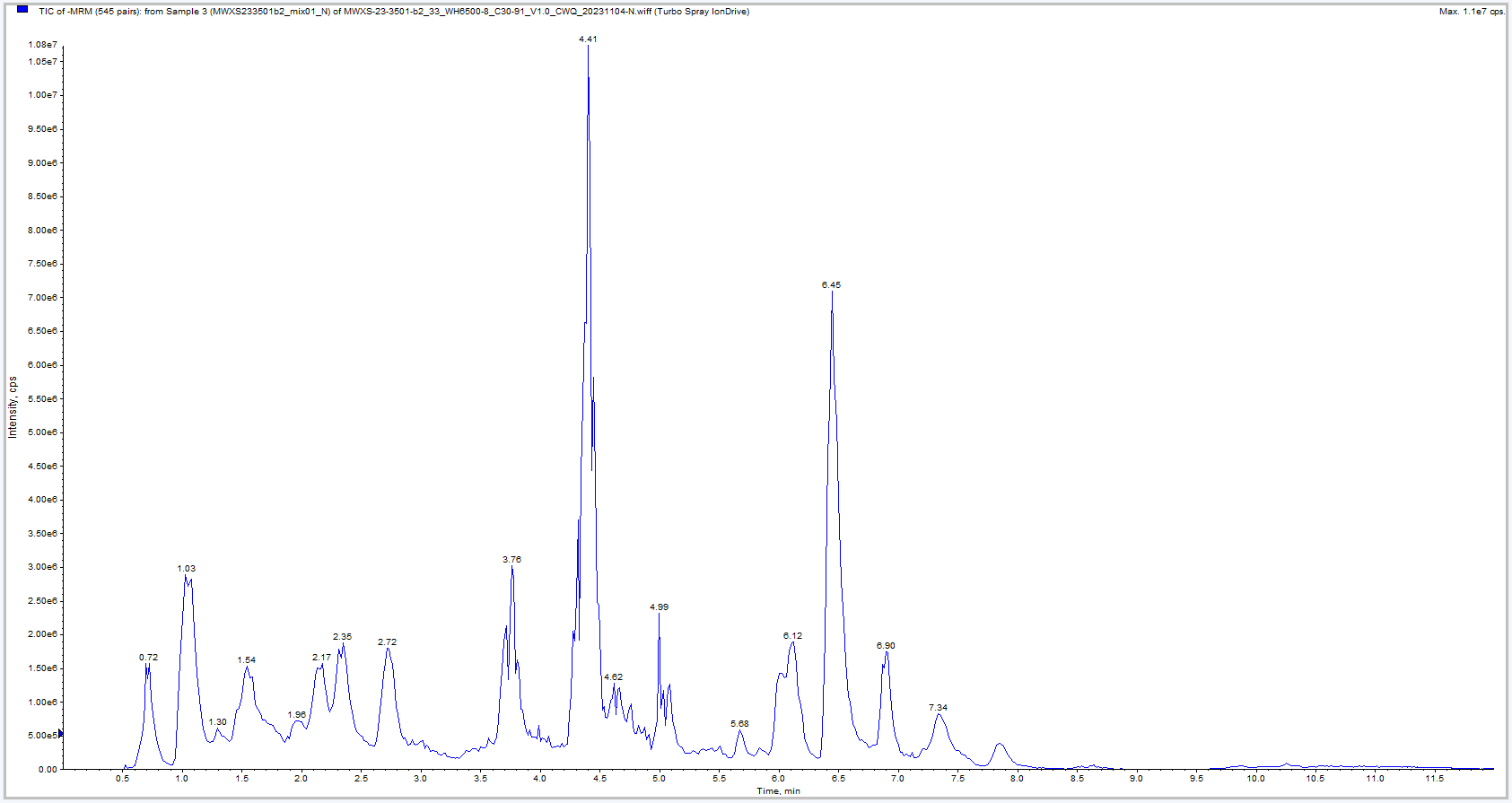

Supplement: Supplementary file 1 [file foods-13-03039-s001.zip › 0.Data/QC/MWXS-23-3501-b2_QC_MS_TIC-N.png]

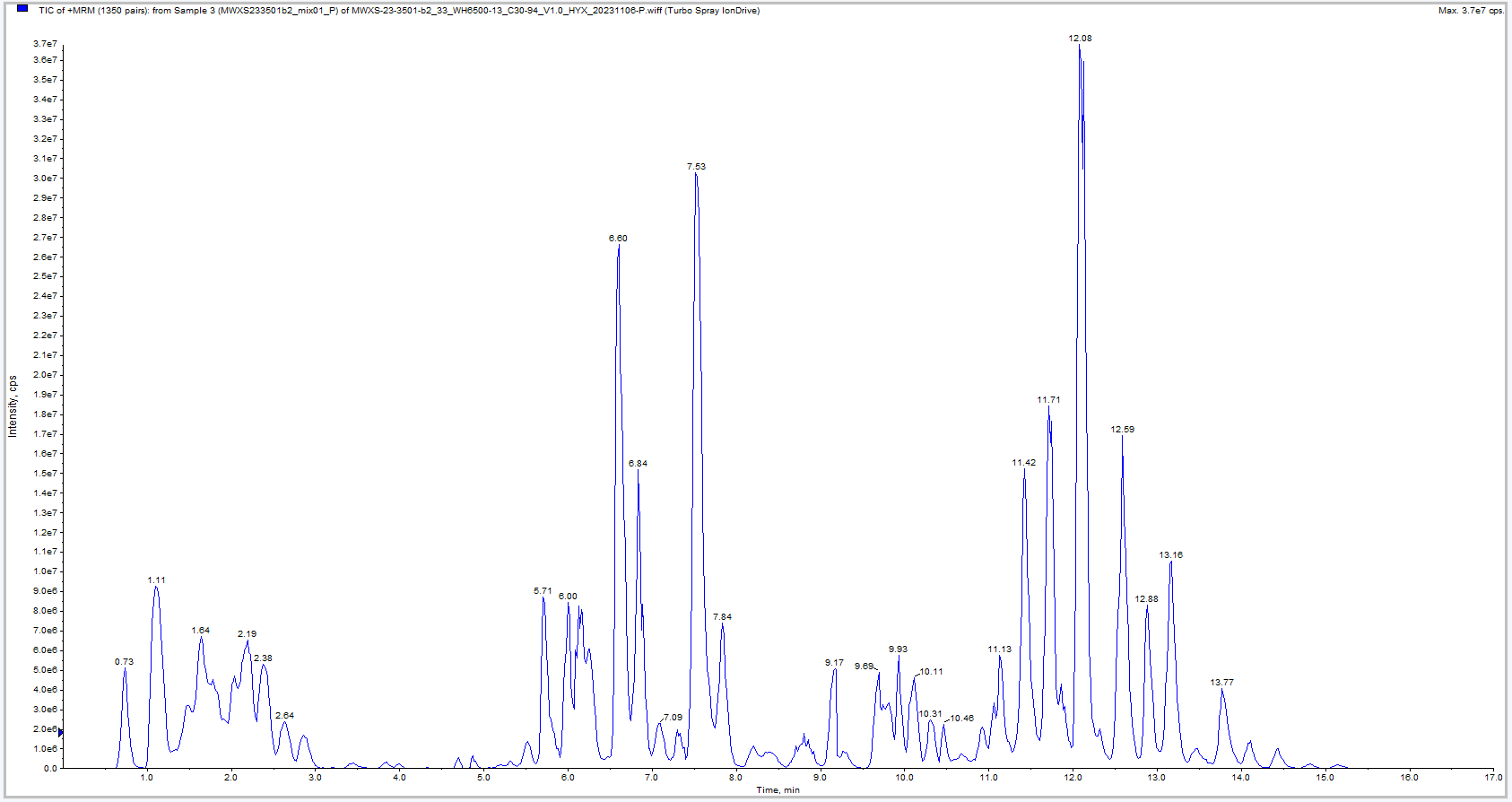

Supplement: Supplementary file 1 [file foods-13-03039-s001.zip › 0.Data/QC/MWXS-23-3501-b2_QC_MS_TIC-P.png]

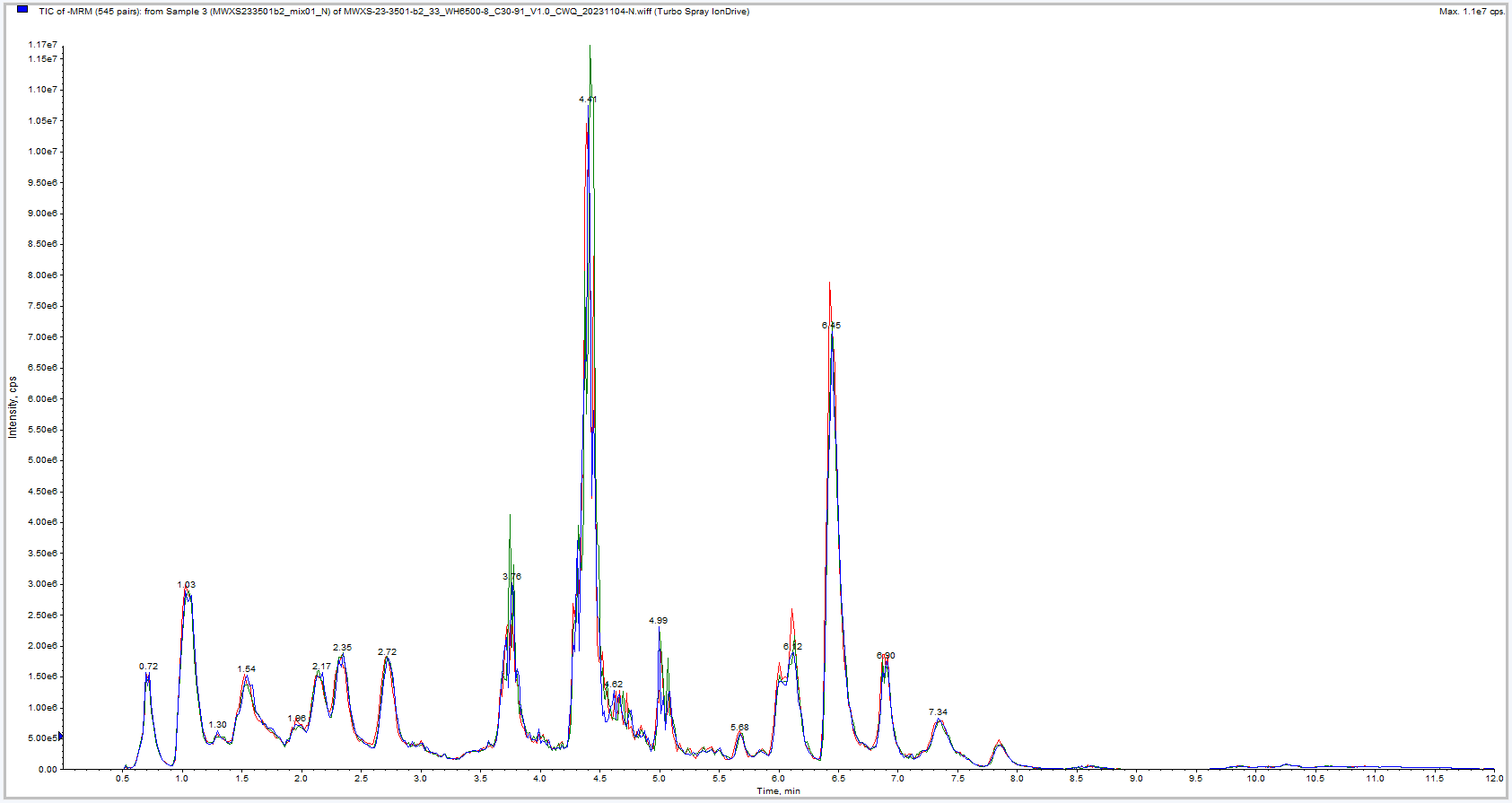

Supplement: Supplementary file 1 [file foods-13-03039-s001.zip › 0.Data/QC/MWXS-23-3501-b2_QC_MS_tic_overlap-N.png]

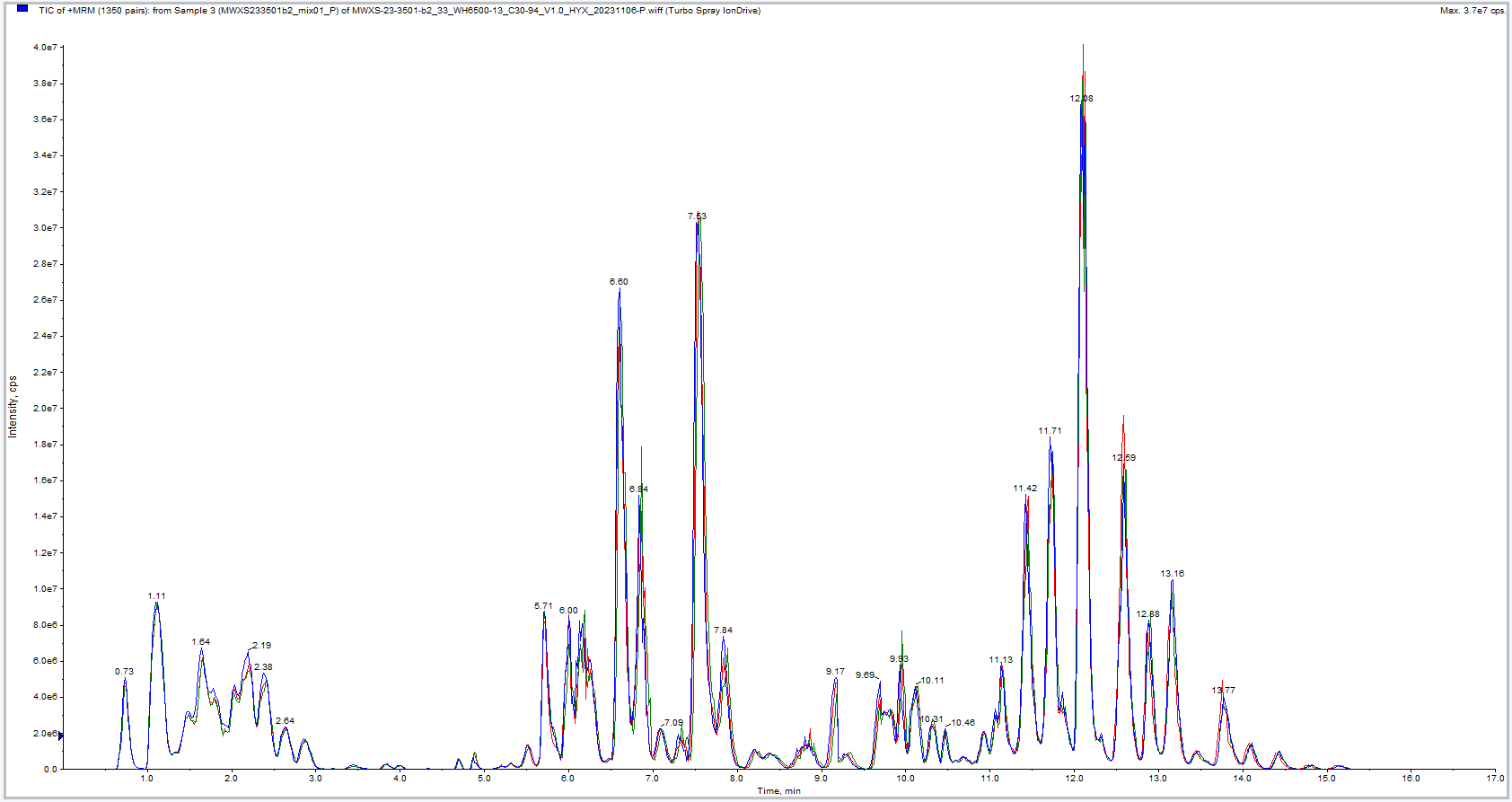

Supplement: Supplementary file 1 [file foods-13-03039-s001.zip › 0.Data/QC/MWXS-23-3501-b2_QC_MS_tic_overlap-P.png]
